# Dysregulated Redox Regulation Contributes to Nuclear EGFR Localization and Pathogenicity in Lung Cancer

**DOI:** 10.1038/s41598-019-41395-8

**Published:** 2019-03-19

**Authors:** Andrew C. Little, Milena Hristova, Loes van Lith, Caspar Schiffers, Christopher M. Dustin, Aida Habibovic, Karamatullah Danyal, David E. Heppner, Miao-Chong J. Lin, Jos van der Velden, Yvonne M. Janssen-Heininger, Albert van der Vliet

**Affiliations:** 1 Department of Pathology and Laboratory Medicine, Robert Larner, M.D. College of Medicine, University of Vermont, Burlington, VT 05405 USA; 2 Present Address: Rogel Cancer Center, Department of Internal Medicine Hematology-Oncology, University of Michigan, Ann Arbor, MI USA; 3 Present Address: Department of Cancer Biology, Dana-Farber Cancer Institute, Boston, MA 02215, USA. Department of Biological Chemistry and Molecular Pharmacology, Harvard Medical School, Boston, MA 02115 USA

## Abstract

Lung cancers are frequently characterized by inappropriate activation of epidermal growth factor receptor (EGFR)-dependent signaling and epigenetic silencing of the NADPH oxidase (NOX) enzyme DUOX1, both potentially contributing to worse prognosis. Based on previous findings linking DUOX1 with redox-dependent EGFR activation, the present studies were designed to evaluate whether DUOX1 silencing in lung cancers may be responsible for altered EGFR regulation. In contrast to normal epithelial cells, EGF stimulation of lung cancer cell lines that lack DUOX1 promotes EGF-induced EGFR internalization and nuclear localization, associated with induction of EGFR-regulated genes and related tumorigenic outcomes. Each of these outcomes could be reversed by overexpression of DUOX1 or enhanced by shRNA-dependent DUOX1 silencing. EGF-induced nuclear EGFR localization in DUOX1-deficient lung cancer cells was associated with altered dynamics of cysteine oxidation of EGFR, and an overall reduction of EGFR cysteines. These various outcomes could also be attenuated by silencing of glutathione *S*-transferase P1 (GSTP1), a mediator of metabolic alterations and drug resistance in various cancers, and a regulator of cysteine oxidation. Collectively, our findings indicate DUOX1 deficiency in lung cancers promotes dysregulated EGFR signaling and enhanced GSTP1-mediated turnover of EGFR cysteine oxidation, which result in enhanced nuclear EGFR localization and tumorigenic properties.

## Introduction

Lung cancer remains to be the leading cause of cancer-related mortality, claiming roughly 1.6 million lives annually worldwide and nearly 150,000 alone in the United States^1,2^. Non-small cell lung cancers (NSCLC) comprise the majority (ca. 80%) of all lung cancers, and are of epithelial origin and include squamous cell carcinomas and adenocarcinomas^3^. A common feature of cancers, including NSCLC, is dysregulated cellular redox metabolism, which is thought to contribute to oncogene addiction and increased invasiveness and malignancy^4,5^. One aspect of such dysregulation is the frequent downregulation of the NADPH oxidase (NOX) family member DUOX1 in many lung cancers, largely by epigenetic mechanisms^6,7^. In the healthy lung epithelium, DUOX1 plays a critical role in innate host defense against injurious triggers and non-microbial pathogens, which is largely mediated by H_2_O_2_ production and redox-dependent activation of protein tyrosine kinases including the epidermal growth factor receptor (EGFR)^8^. Recent studies suggest that DUOX1 silencing in cancers is associated with worse prognosis, and that loss of epithelial DUOX1 promotes features of epithelial-to-mesenchymal transition (EMT), invasive properties, as well as enhanced cancer stem-cellness and resistance to the EGFR tyrosine kinase inhibitor erlotinib^5,9^.

Dysregulated EGFR signaling is a well-appreciated component of most epithelial cancers, which is manifested by increased expression and activation of this kinase. In some cases, this is related to EGFR-activating mutations (e.g. L858R, exon 19 del, etc.), which has fueled the design of molecular-based therapeutics, such as EGFR tyrosine kinase inhibitors, although this has been complicated by the development of acquired resistance due to secondary EGFR mutations or alternative resistance mechanisms^10–12^, and has resulted in development of next generation EGFR-targeted therapies^13–15^. Another aspect of dysfunctional EGFR regulation in cancer is its altered subcellular distribution and protein turnover. Following ligand binding, EGFR is typically endocytosed via clathrin-dependent and -independent mechanisms, followed by endosomal recycling back to the cell surface, lysosomal degradation, or transport to various intracellular organelles^16,17^. EGFR endocytic homeostasis is often dysregulated in various cancers, leading to irregular trafficking to intracellular organelles, specifically the nucleus. Nuclear EGFR localization is mediated by retrograde ER transport involving importin and Sec. 61 translocon^18,19^, and is dependent on EGFR phosphorylation on Y1101 by Src-family kinases^20,21^. EGFR protein localized within the nucleus (nEGFR) has been detected in many cancers, including NSCLC, and promotes survival by functioning as a nuclear kinase that enhances DNA replication and repair and by acting as a co-transcriptional activator by kinase-independent interaction with transcription factors such as STAT3 to regulate genes involved in tumor progression^22^. Moreover, nEGFR has been associated with poor overall survival in NSCLC^23^, as well as enhanced resistance to EGFR targeted therapies^23,24^, particularly because it may involve EGFR actions independent of tyrosine kinase function.

Pioneering studies by Carroll and co-workers revealed the importance of a redox-based mechanism in regulating EGFR tyrosine kinase function, involving sulfenylation of C797 in the ATP-binding region of the kinase domain^25,26^. Molecular studies indicate that sulfenylation of C797 (ie. conversion to R-SOH) can enhance kinase function, and that subsequent conjugation with GSH (to form *S*-glutathionylated EGFR; R-SSG) is likely responsible for restoring inactive EGFR and preventing further irreversible oxidation^26,27^. Moreover, such EGFR sulfenylation is mediated by activation of NOX isoforms including DUOX1, especially in the context of innate epithelial responses to common airborne triggers^27,28^. Although previous studies have suggested alterations in redox-mediated EGFR regulation in lung cancer^26^, it is unclear how suppression of DUOX1 in lung cancer impacts on EGFR regulation. Here, we demonstrate a direct association between DUOX1 silencing and nEGFR function in lung cancers, and increased pathogenicity and resistance to cetuximab. Moreover, nEGFR localization and function are linked to altered dynamics of reversible cysteine oxidation within EGFR, specifically with increased turnover of cysteine oxidation and formation of reduced EGFR, suggesting that reduction of oxidized C797 is critical for its nuclear kinase-independent function(s) as a co-transcriptional regulator. Finally, we demonstrate that such turnover of cysteine oxidation and nEGFR function depends on the presence of glutathione S-transferase P 1 (GSTP1), a known catalyst of GSH conjugation to oxidized cysteines and an important determinant of metabolic dysfunction and pathogenicity in cancer.

## Results

### DUOX1 status in lung cancer cells affects EGF-induced EGFR internalization and nuclear localization

**Figure 1:**
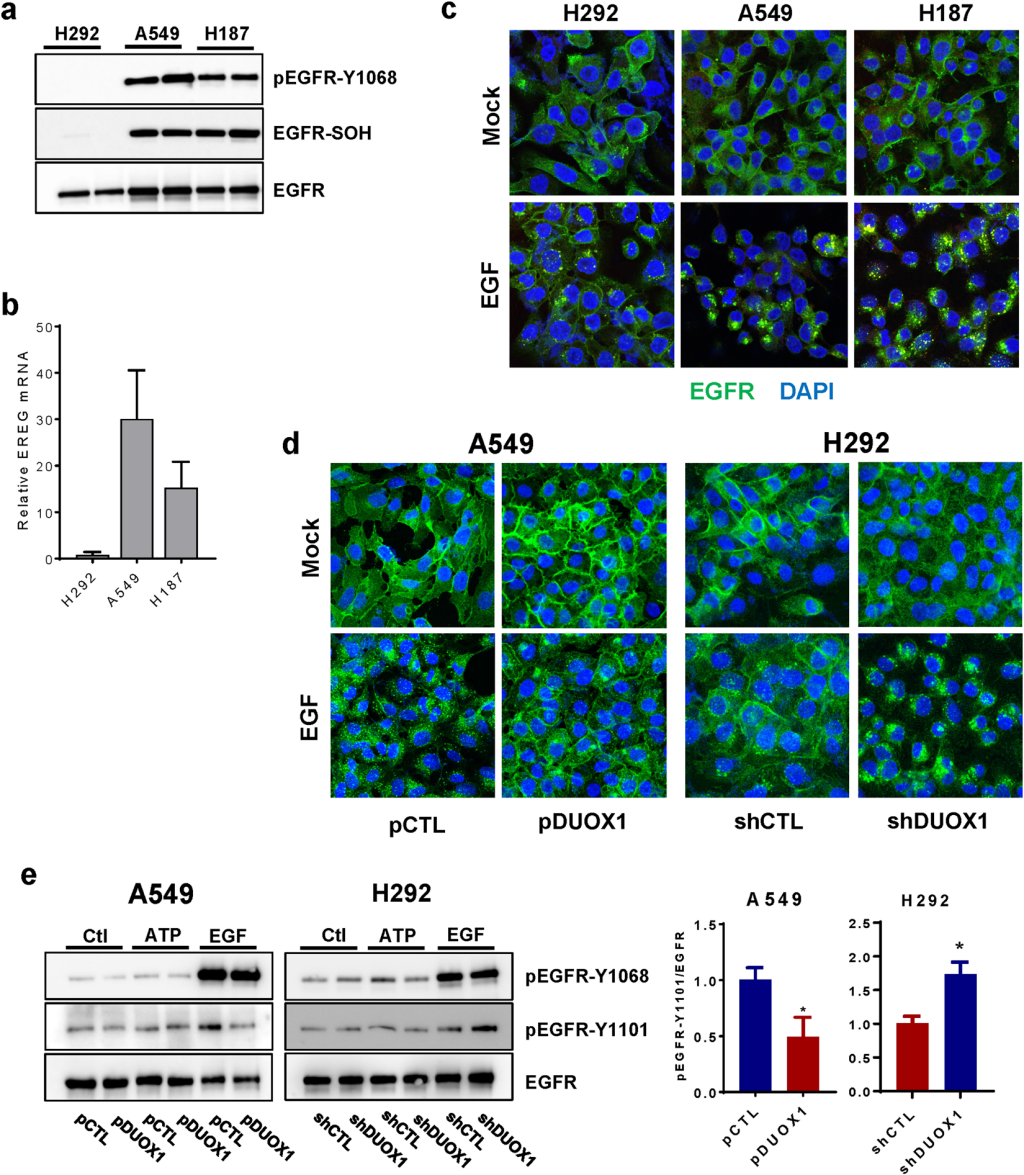
EGFR activation and EGFR intracellular trafficking is altered in cancer cells that lack DUOX1. (**a**) Representative western blot analysis of EGFR autophosphorylation (Y1068) and cysteine oxidation (EGFR-SOH) in DUOX1-containing H292 cells or DUOX1-deficient A549 or H187 cells, after overnight culture under serum-starved conditions. Blots are representative of at least 2 independent experiments. (**b**) Analysis of EREG mRNA in various cancer cell lines. (2 separate experiments in triplicate). (**c**) Analysis of EGFR localization (green) by immunofluorescence imaging, showing localization primarily at the plasma membrane in mock-treated cells, and marked internalization of EGFR in A549 and H187 cells in response to EGF (100 ng/mL; 20 min). Blue: nuclear DAPI stain. (**d**) EGF-induced EGFR internalization is prevented by DUOX1 overexpression (A549-pDUOX1) and enhanced by DUOX1 silencing (H292-shDUOX1). Results are representative images of three experiments. (**e**) Western blot analysis of EGFR phosphorylation at Y1068 or Y1101 in A549 or H292 cells (either expressing or lacking DUOX1), in response to 20 min stimulation with ATP (100 µM) or EGF (100 ng/mL). Densitometry analysis of EGF-induced EGFR-Y1101 phosphorylation of different cell models is shown at right (^*^p < 0.05 compared to corresponding control; n = 4; t-test).

Based on previous studies indicating DUOX1 silencing in lung cancers and its impact on resistance to EGFR tyrosine kinase inhibitors^9^, we evaluated basal activity and subcellular localization of EGFR within various lung cancer cell lines in relation to DUOX1 expression. We chose to focus on 4 lung cancer cell lines, H292, A549, H187, and H460, which all express EGFR at comparable levels and are not known to contain any EGFR mutations^9^. Among these, H292 cells possess epithelial characteristics and express DUOX1 at levels similar to normal bronchial epithelial cells, whereas the other 3 cell lines display more mesenchymal and invasive features, to various degrees, and have low or almost undetectable DUOX1 expression^9^ **(**Supplementary Fig. S1**)**. Under serum-starved conditions, lung cancer cell lines that lack DUOX1 (A549, H187) show detectable basal EGFR activity (as assessed by Y1068 autophosphorylation, a read-out of EGFR kinase activation), whereas this is largely absent in normal bronchial epithelial cells^28^ or H292 cells (with epithelial characteristics) that express DUOX1 (Fig. 1a). This likely reflects autocrine EGFR stimulation, as supported by increased expression of EGF ligands such as epiregulin (EREG) in lung cancer cells (Fig. 1b). Overall EGFR expression and distribution was not markedly different between these different cancer cells, and EGFR was largely localized to the plasma membrane (Fig. 1c). However, whereas EGFR remained mainly localized at the plasma membrane in H292 cells after stimulation with EGF (100 ng/mL; 20 min), most likely as a result of effective recycling, similar EGF stimulation of DUOX1-deficient A549 cells or H187 cells resulted in marked EGFR internalization, as indicated by punctate intracellular staining and apparent perinuclear localization (Fig. 1c). To determine whether such alterations in EGFR localization were directly related to DUOX1 status, we confirmed that EGF-induced internalization was largely prevented in A549 cells (Fig. 1d) after DUOX1 overexpression. Conversely, EGF-induced similar EGFR internalization in H292 cells after DUOX1 silencing (Fig. 1d). Collectively, these observations indicate that DUOX1 status impacts on EGFR internalization and/or trafficking dynamics, and appears to affect nuclear EGFR localization.

**Figure 2:**
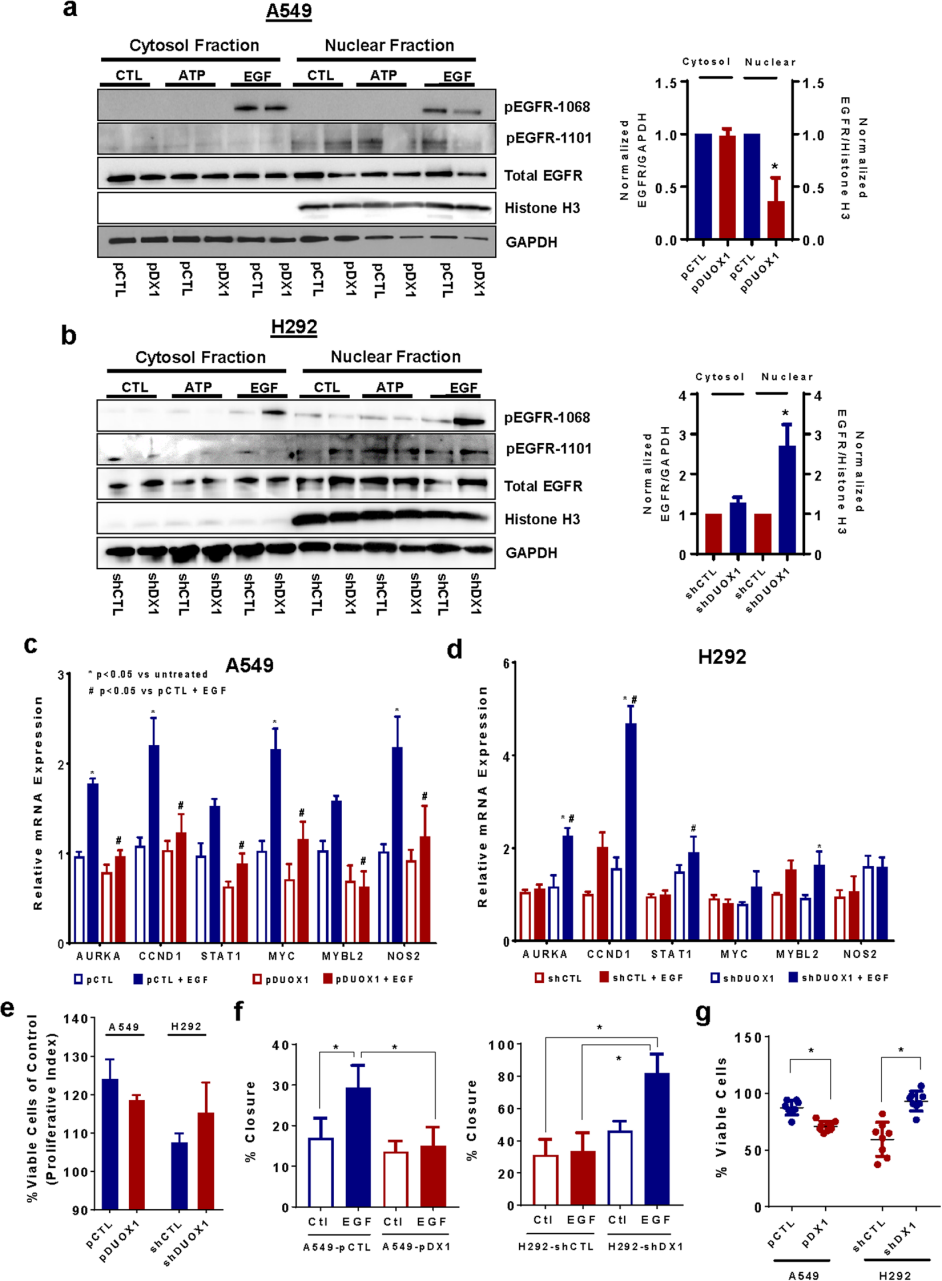
DUOX1 status determines EGF-induced nuclear EGFR localization and related functional outcomes. (**a,b**) Western blot analysis of subcellular fractions of A549 cells (**a**) or H292 (**b**) in which DUOX1 was overexpressed or silenced (pDX1 vs pCTL; shDX1 vs. shCTL) after stimulation with ATP (100 µM) or EGF (100 ng/mL) for 20 min. Bar graphs on the right show densitometry analysis of EGFR in cytosolic and nuclear fractions in EGF-stimulated cells (^*^p < 0.05; n = 4; t-test). (**c,d**) RT-qPCR analysis of genes regulated by nEGFR in unstimulated or EGF-stimulated (100 ng/mL, 1 hr) A549 cells (**c**) or H292 cells (**d**), in which DUOX1 was either overexpressed (pDUOX1) or silenced (shDUOX1). ^*^p < 0.05 compared to unstimulated control; ^#^p < 0.05 compared to corresponding control cell model (pCTL or shCTL); n = 4; one-way ANOVA. (**e**) Analysis of cell proliferation of various cell models (n = 4, from 2 separate experiments). (**f**) Analysis of EGF-stimulated wound closure in scratch assays of A549 cell models (left) or H292 cell models (right). ^*^p < 0.05; n = 8; one-way ANOVA. (**g**) Effect of DUOX1 overexpression or silencing on sensitivity to anti-EGFR-antibody based inhibition (^*^p < 0.01; n = 8; two-tailed t-test).

Further indicative of nuclear EGFR translocation, EGF stimulation of A549 or H187 cells resulted in enhanced EGFR phosphorylation at Y1101 on the C-terminal tail of EGFR, a critical feature in nuclear EGFR trafficking^21^, which was attenuated after DUOX1 overexpression **(**Fig. 1e; Supplementary Fig. S2). Conversely, EGF stimulation of H292 cells promoted Y1101 phosphorylation only after DUOX1 was silenced (Fig. 1e). Time course studies indicated that EGF-induced Y1101 phosphorylation in A549 cells occurs rapidly (within 1 min) and is both delayed and attenuated after DUOX1 overexpression (Supplemental Fig. S3), consistent with the notion that Y1101 phosphorylation precedes nuclear EGFR localization. In contrast, DUOX1 status did not affect overall EGF-induced EGFR activation as reflected by autophosphorylation at Y1068 (Fig. 1e). Also, EGFR transactivation by cell stimulation with ATP (100 µM; 20 min), which is mediated by DUOX1^29^, did not significantly increase pEGFR-Y1101 (Fig. 1e). Nuclear EGFR localization in response to EGF in DUOX1-deficient cancer cells was further verified by subcellular fractionation, showing that EGF stimulation increased the presence of EGFR (and its phosphorylated forms pEGFR-Y1068 and pEGFR-Y1101) in nuclear extracts of A549 cells, which was suppressed after DUOX1 overexpression (Fig. 2a). Conversely, EGF-induced increases in nEGFR and pEGFR-Y1068 and pEGFR-Y1101 were observed in H292 cells only after DUOX1 silencing (Fig. 2b). Nuclear EGFR localization was confirmed by immuno-EM labeling (**Supplemental Fig S4**).

### nEGFR localization in DUOX1-deficient cancer cells is related to increased expression of nEGFR-regulated genes and increased tumorigenic properties

Nuclear EGFR (nEGFR) can act as a transcriptional co-activator for various oncogenic genes involved in proliferation and cell cycle progression, such as aurora kinase (AURKA), cell cycle dependent kinase 1 (CCND1), signal transducer and activator of transcription 1 (STAT1), MYC proto-oncogene, MYB proto-oncogene like 2 (MYBL2), and inducible nitric oxide synthase (NOS2)^22^. We therefore evaluated whether DUOX1 status affects EGF-induced expression of these nEGFR-regulated genes, and indeed observed that EGF was capable of inducing most of these genes in A549 cells, whereas this was significantly attenuated after DUOX1 overexpression (Fig. 2c). Conversely, induction of nEGFR-regulated genes was observed in H292 cells only after DUOX1 silencing (Fig. 2d). To evaluate the impact of DUOX1 status for functional outcomes associated with nEGFR, we evaluated EGF-dependent cell proliferation and cell migration. EGF-stimulated proliferation was consistently greater in DUOX1-deficient H292 or A549 cells, although differences were not statistically significant (Fig. 2e). Moreover, the ability of EGF to enhance cell migration in serum-free media was observed only in DUOX1-deficient cells (Fig. 2f, and Supplemental Fig. 5), consistent with previous findings linking DUOX1 silencing to increased epithelial-to-mesenchymal transition and cancer cell invasiveness^9^. Finally, since nEGFR can promote resistance against anti-EGFR antibodies^24^, we evaluated anti-EGFR resistance after DUOX1 silencing or overexpression, which consistently demonstrated increased resistance in A549 cells that lack DUOX1 (Fig. 2g; Supplemental Fig. S6). In addition, we previously demonstrated that DUOX1 silencing can also promote resistance against EGFR tyrosine kinase inhibitors such as erlotinib^9^. Collectively, these findings indicate DUOX1 silencing in lung cancer cells impacts on EGFR internalization and nuclear translocation in response to EGF, with corresponding consequences for EGFR-mediated cell proliferation and migration, as well as sensitivity to (antibody-based) EGFR targeting.

### DUOX1-deficiency in lung cancer cells affect dynamics of EGFR redox regulation

**Figure 3:**
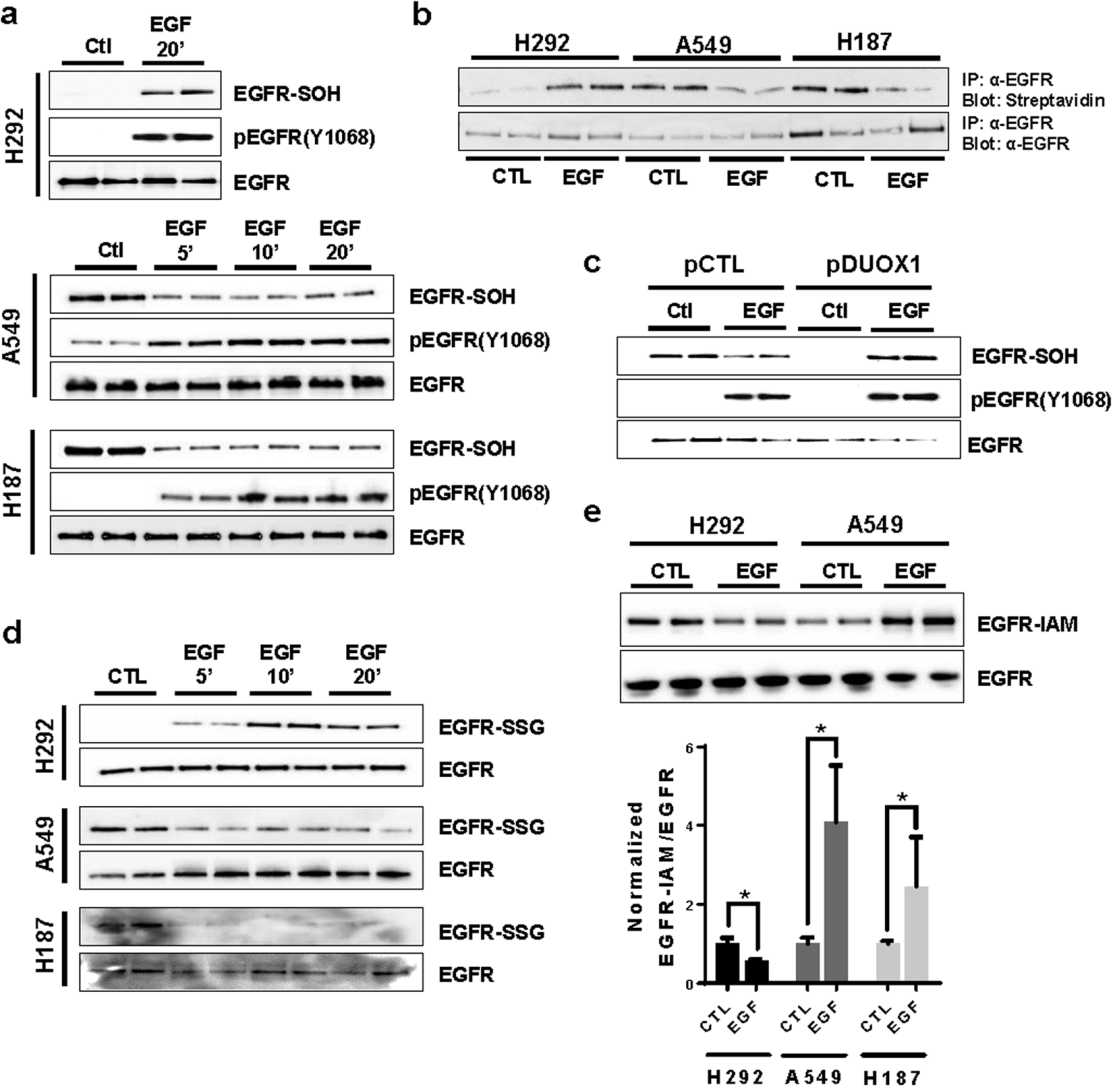
EGFR cysteine oxidation dynamics is altered in lung cancer cells. (**a**) Analysis of basal and EGF-dependent EGFR autophosphorylation (pY1068) and sulfenylation (EGFR-SOH; measured by DCP-Bio1 labeling and analysis of avidin-purified proteins) in various cell lines. All blots are representative of at least 2 independent experiments. (**b**) EGFR was immunoprecipitated from DCP-Bio1-derivatized cell lysates and analyzed by streptavidin blotting or α-EGFR. Representative of 2 independent experiments. (**c**) Effect of DUOX1 overexpression on basal and EGF-dependent EGFR autophosphorylation (pY1068) and sulfenylation (EGFR-SOH) in A549 cells. Representative of 2 independent experiments. (**d**) Western blot analysis of basal and EGF-dependent EGFR S-glutathionylation (EGFR-SSG) in various cancer cell lines. Representative of 2 independent experiments. (**e**) Western blot analysis of EGFR cysteine thiols by BIAM labeling (EGFR-IAM) in H292 and A549 cells. Bar graph shows quantified densitometry analysis from 4–6 replicates from 2–3 separate experiments in H292, A549 and H187 cells (^*^p < 0.05, t-test). Blots are representative of at least 2 independent experiments.

**Figure 4:**
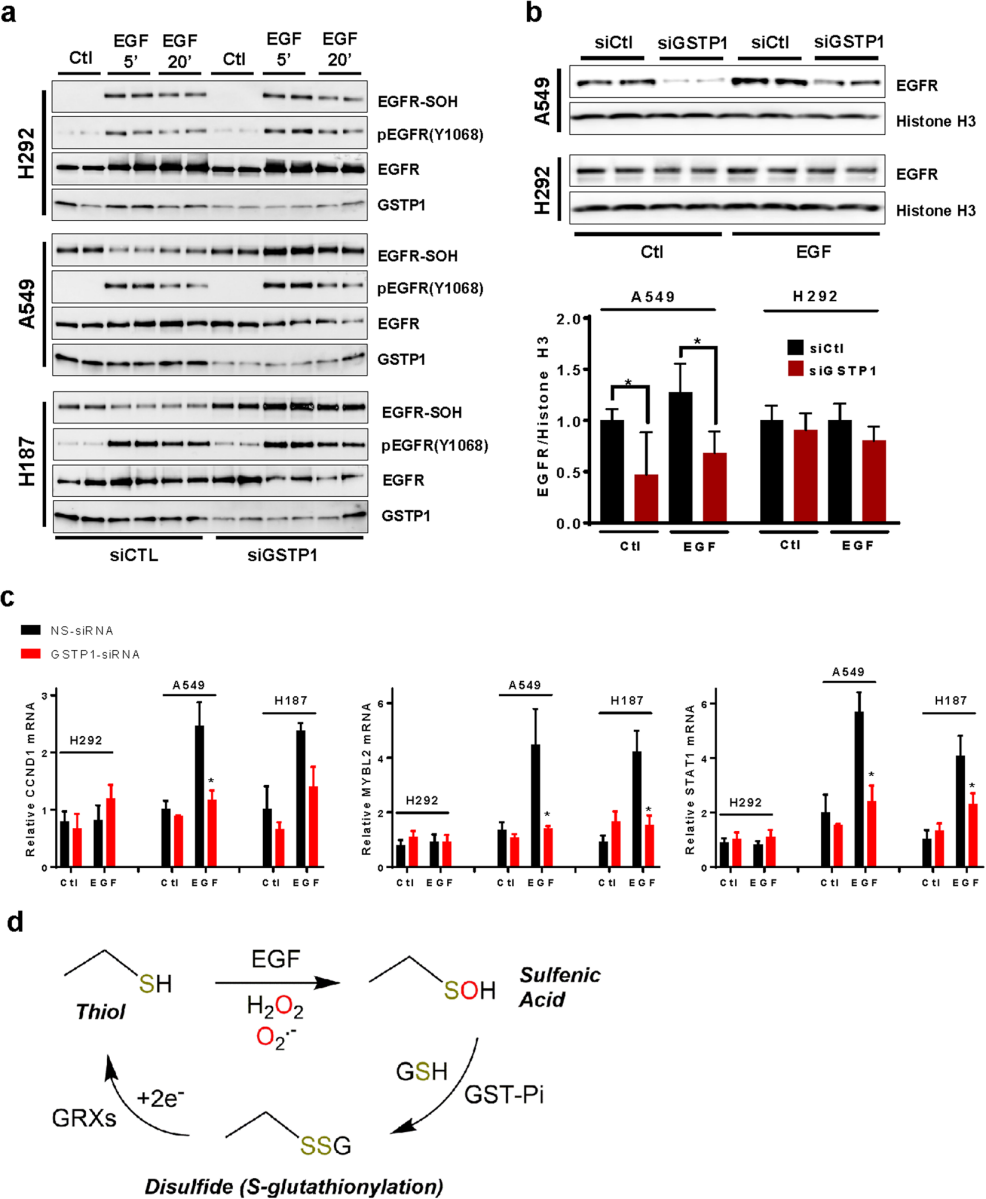
Altered EGFR oxidation and nuclear EGFR localization in lung cancer cells depends on GSTP1. (**a**) Analysis of EGF-induced EGFR cysteine oxidation and autophosphorylation in cancer cell lines after GSTP1 silencing by siRNA. Western blots are representative of at least 2 independent experiments. (**b**) Western blot analysis of EGFR and Histone H3 in nuclear extracts of untreated or EGF-treated cancer cells after siRNA silencing of GSTP1. Bar graph represents quantified data from densitometry analysis of 2 independent experiments in duplicate (^*^p < 0.05, n = 4; t-test). (**c**) RT-qPCR analysis of nEGFR-regulated genes after GSTP1 silencing. ^*^p < 0.05 by two-way ANOVA and Sidak’s multiple comparisons test (n = 3–5). (**d**) Schematic of EGFR cysteine oxidation and proposed regulation by GSTP1 and reducing systems.

Previous studies indicate that EGFR kinase activity can be regulated by oxidation of a conserved cysteine in its kinase domain, C797, to a sulfenic acid (EGFR-SOH)^26–28^. Accordingly, we observed increased basal EGFR sulfenylation in serum-starved A549 or H187 cells (with low DUOX1 expression) compared to DUOX1-expressing H292 cells (Fig. 1a), corresponding to increased EGFR activity. Also, in agreement with previous reports^26,27^, EGF stimulation of DUOX1-expressing H292 cells resulted in increased EGFR autophosphorylation as well sulfenylation (analyzed by labeling with DCP-Bio1 and analysis of avidin-purified proteins; Fig. 3a), both of which are transient and reversed over time^27^ (see also Fig. 4a). However, whereas EGF similarly enhanced EGFR autophosphorylation in A549, H187 cells, or H460 cells, EGFR sulfenylation was *suppressed* in these cases (Fig. 3a; Supplemental Fig. S7a). Such reduction of EGFR sulfenylation occurred rapidly (as early as 5 min after EGF stimulation; Fig. 3a) and was also observed at lower doses of EGF (4–20 ng/mL) (Supplemental Fig. 7b). Indeed, whereas EGF-induced EGFR autophosphorylation corresponded temporally with the extent of EGFR sulfenylation in DUOX1-expressing H292 cells^27^, these events were dissociated in DUOX1-deficient cancer cells (Fig. 3a). EGF-induced changes in EGFR-SOH were confirmed by streptavidin blotting of immunopurified EGFR from cell lysates (Fig. 3b). Notably, these differences in EGFR cysteine oxidation in the various cell models were not associated with significant differences in cellular oxidant status, measured by incubation with redox-sensitive fluorescent probes (Supplemental Fig. S8). Overall, these findings suggest that EGF-induced EGFR internalization and nuclear translocation in DUOX1-deficient cancer cells is associated with altered dynamics of EGFR oxidation. Consistent with this notion, overexpression of DUOX1 in A549 cells, which minimized nuclear EGFR translocation (Fig. 1), resulted in attenuated basal EGFR sulfenylation and enhanced EGF-stimulated EGFR sulfenylation (Fig. 3c), similar to H292 cells (Fig. 3a).

We next wished to address the fate of sulfenylated cysteines, which can either react with cellular GSH to form *S*-glutathionylated cysteines^27,30^ or become further oxidized due to elevated production of reactive oxygen species (ROS) in cancer cells^26^. To address EGFR *S*-glutathionylation (EGFR-SSG), cells were preloaded with biotinylated GSH after which biotin (ie. GSH) incorporation into proteins was monitored. As expected^27^, EGF stimulation led to increased EGFR-SSG levels in H292 cells (Fig. 3d), as a secondary oxidative event following sulfenylation. In contrast, increased basal levels of EGFR-SSG were observed in A549 or H187 cells compared to H292 cells (Supplemental Fig. S9), and EGFR-SSG levels were rapidly depleted in A549 and H187 cells following EGF stimulation (Fig. 3c), indicating that EGF stimulation also enhances turnover of EGFR-SSG in these cases. To address the possibility that EGFR-SOH was further oxidized to sulfinic/sulfonic acids in response to EGF, cells were lysed in the presence of the thiol-specific reagent, iodoacetyl-LC-biotin, to determine the overall reduced thiol content in EGFR (EGFR-SH). As expected^29^, EGF stimulation of H292 cells resulted in reduced EGFR-SH content, in agreement with observed increases in EGFR sulfenylation and *S*-glutathionylation. However, EGFR-SH content was *enhanced* after similar stimulation of A549 and H187 cells (Fig. 3e, Supplemental Fig. S10), suggesting that loss of EGFR-SOH or EGFR-SSG in response to EGF was not associated with increased (irreversible) cysteine oxidation, but was instead associated with overall reduction of oxidized cysteines within EGFR. Collectively, these various findings suggest that EGF stimulation results in accelerated turnover of cysteine oxidation of EGFR in DUOX1-deficient A549 and H187 cells, potentially due to enhanced conversion to EGFR-SSG and subsequent reduction to EGFR-SH.

### Dysregulated EGFR cysteine oxidation and nuclear targeting is mediated by GSPT1

Although EGF-stimulated EGFR cysteine sulfenylation has been associated with increase kinase activation and EGFR autophosphorylation, we speculated that subsequent modifications such as *S*-glutathionylation might be responsible for EGFR internalization and nuclear localization. To test this, we aimed to prevent EGFR *S*-glutathionylation in cancer cells by inhibition of glutathione *S*-transferase P1 (GSTP1), a known catalyst of protein *S*-glutathionylation^30^ and a potential direct substrate for phosphorylation and activation by EGFR^31^. GSTP1 is also frequently overexpressed in solid tumors and may contribute to malignancy and drug resistance^32,33^. Somewhat surprisingly, GSTP1 silencing using siRNA did not significantly affect EGF-induced EGFR-SOH levels in H292 cells, perhaps because DUOX1 activation somehow resulted in local inactivation of GSTP1, but was found to markedly prevent EGF-induced loss of EGFR-SOH in both A549 and H187 cells (Fig. 4a). Importantly, these alterations in EGFR cysteine oxidation did not impact on overall EGFR activation (pEGFR-1068). In addition, siRNA silencing of GSTP1 in A549 cells was also found to attenuate EGF-induced nEGFR localization (Fig. 4b), and induction of nEGFR-regulated genes (Fig. 4c), whereas no such effects were observed in H292 cells (Fig. 4b,c and Supplemental Fig. S11). Collectively, these findings suggest that altered EGFR internalization in DUOX1-deficient lung cancer cells is associated with enhanced GSTP1-mediated *S-*glutathionylation of EGFR-SOH and subsequent reduction to EGFR-SH, whereas such turnover of EGFR-SOH is delayed in DUOX1-expressing cells and is independent of GSTP1 (Fig. 4d). Furthermore, such altered turnover and reduction of EGFR may contribute to its post-endocytic processing, nuclear transport, and/or non-kinase functions as a co-transcriptional regulator.

## Discussion

Nuclear translocation of EGFR has recently been appreciated to strongly correlate with oncogenic outcomes in various cancers, involving both kinase-dependent and –independent functions of nEGFR, and further characterization of the molecular events involved in spatial regulation of EGFR are critically important in the development of novel therapeutics^34^. In this work, we present evidence that the extent of nEGFR localization in lung cancer cells is determined by the presence or absence of DUOX1, an NADPH oxidase homolog that is important for normal epithelial function and is closely associated with regulating EGFR-dependent innate responses to infection or injury^28,35^. The present studies also extend our recent findings indicating strong association of DUOX1 silencing in lung cancers with functional oncogenic properties, such as epithelial-to-mesenchymal transition, cancer stem cell properties, and resistance to EGFR kinase inhibitors^9^. Finally, our studies highlight the importance of dynamic redox-dependent regulation of EGFR for both tyrosine kinase function as well as internalization and nuclear translocation, and indicate that loss of DUOX1 in lung cancers results in altered dynamics of such redox regulation, thereby favoring nuclear EGFR functions.

The importance of redox-dependent events in regulating EGFR signaling is well appreciated^36^, but it was only recently recognized that EGFR can be directly regulated by redox-dependent modification of a conserved cysteine (C797) within it kinase domain^25^. Molecular studies subsequently demonstrated that oxidation of C797 to a sulfenic acid enhances intrinsic tyrosine kinase activity^26,27^, although alternative redox-dependent modifications of C797 have also been described with less well-characterized functional consequences^37,38^. Observations that sulfenylation of EGFR is closely associated with EGFR autophosphorylation, both in dose- and time-dependent studies, support such as causal relationship between cysteine sulfenylation and EGFR kinase activation^26,27^. However, the present studies indicate that these events are not necessarily tightly linked and suggest potential alternative functions of EGFR cysteine oxidation in regulating subcellular trafficking and kinase-independent functions. Indeed, alternative cysteine modifications have been linked to altered subcellular distribution of several proteins^39,40^. Our observation of reduced EGFR sulfenylation in response to EGF in various cancer cells (A549, H187), in spite of increased EGFR kinase activation, appears to be at odds with previous studies by Truong and coworkers who demonstrated that brief EGF stimulation (2 min) of A549 cells also enhanced EGFR sulfenylation^26^, whereas our results indicated a loss of sulfenylated EGFR as early as 5 min after EGF stimulation. Collectively, these findings indicate that the dynamics of EGFR cysteine oxidation and its reversal, which occurs over a 20–30 min time scale in normal epithelial cells or in H292 cells^27^, are dramatically altered and occur at much shorter time scales in cancer cells most likely because they typically have enhanced resistance to redox alteration due to e.g. Nrf2 addition^41^. Importantly, these altered dynamics appear to be critical for controlling EGFR subcellular trafficking and for nuclear (kinase-independent) functions of EGFR, since these events could be attenuated by suppression of GSTP1, an important enzyme in controlling cysteine oxidation and turnover^30,42^. Moreover, our findings also offer an alternative explanation how GSTP1 may contribute to pro-cancerous features, as a potential substrate for EGFR after its internalization^31^. Intriguingly, studies of primary gliomas indicated nuclear localization of GSTP1 which was inversely correlated with survival^32^, suggesting that nuclear localization of GSTP1 as well as EGFR might be coordinated. Finally, our findings suggest that reduction of EGFR cysteines to its reduced thiol may be critical for its co-transcriptional function, analogous to redox regulation of other transcription factors such as e.g. NF-κB and STAT3 by e.g. thioredoxins that are commonly overexpressed in cancers^43,44^.

Our observation that accelerated turnover of cysteine oxidation in lung cancers is associated with the relative absence of DUOX1, and can in fact be attenuated by DUOX1 overexpression, suggests that DUOX1 plays an important role in prolonged cysteine oxidation in normal epithelial cells to mediate appropriate EGFR activation and recycling. In contrast, in cancer cells that lack DUOX1, abnormal dynamics of EGFR cysteine oxidation are associated with aberrant EGFR trafficking and nuclear localization, although the precise molecular mechanism(s) by which this occurs remain unclear. Such a proposed role for DUOX1 in regulating normal EGFR turnover is supported by findings that EGF-induced activation of EGFR can activate DUOX1^45^. However, DUOX1 does not appear to play a significant role in regulating EGFR activation in this context based on EGFR autophosphorylation^27^, which appears to be primarily mediated by redox-dependent regulation of EGFR autophosphorylation by a different NADPH oxidase, NOX2^25,27^. In fact, such NOX2-dependent mechanisms are likely responsible for increased EGFR cysteine oxidation in cancer cell lines under basal conditions, due to constitutive activation of EGFR ligands such as EREG (Fig. 1). This is in apparent contrast to EGFR transactivation by e.g. ATP-mediated purinoceptor activation, in which DUOX1 plays a prominent role, by promoting redox-dependent activation of the non-receptor tyrosine kinase Src and shedding of EGFR ligands^29^. Based on these considerations, we propose that DUOX1 activation during ligand-dependent EGFR activation may play an important role in regulating EGFR internalization and recycling, which may therefore be disturbed in cancer cells that lack DUOX1, in favor of nuclear EGFR localization.

The functional consequences of EGFR activation are strongly dictated by receptor endocytosis, which occurs through both clathrin-dependent and -independent mechanisms, and results in either receptor degradation, recycling to the plasma membrane, or altered EGFR distribution towards nuclei or mitochondria^16,34^. Clathrin-independent EGFR endocytosis has been associated with receptor ubiquitination and lysosomal degradation^46^, but clathrin-dependent mechanisms contribute to intracellular EGFR trafficking to the nucleus^47^ and chemotactic tumor invasion^48^. Moreover, both clathrin-dependent and -independent EGFR internalization dynamics are regulated by Ca^2+^-dependent processes and/or protein kinase C^49,50^, although clathrin-dependent endocytosis may involve both Ca^2+^-dependent and -independent mechanisms, with disparate effects in endocytosis of different proteins into distinct clathrin structures^49,51^. Intriguingly, since both Ca^2+^ and PKC are also required for activation of DUOX1 by diverse stimuli including EGF^45,52,53^, it is plausible that DUOX1 participates in regulating ligand-induced EGFR activation by affecting yet to be determined steps involved in e.g. clathrin-dependent endocytosis pathways and/or subsequent nuclear EGFR trafficking, by regulating cysteine oxidation of EGFR or other protein targets involved in such processes^54^. An intriguing recent study indicated that EGF-mediated activation of DUOX1 within the Golgi complex causes transient inactivation of the phosphatidylinositol 4-phosphate [PtdIns(4)P] phosphatase Sac1 by cysteine oxidation, leading to local accumulation of PtdIns(4)P^55^. PtdIns(4)P plays an important role in the *trans*-Golgi network in regulating membrane traffic, but also mediates receptor sorting at early endosomes, including EGFR^56^. Hence, downregulation of DUOX1 in lung cancers could lead to altered EGFR internalization pathways, and could thereby promote nuclear EGFR targeting and associated tumorigenic functions.

It is important to discuss several limitations and remaining questions resulting from our studies. First, we presume that the observed cysteine oxidations in our studies apply primarily to C797 within the ATP-binding region of the EGFR kinase domain^26,37^. Indeed, C797 sulfenylation may affect kinase function, but C797 is not essential for EGFR kinase activity^26,57^. Also, C797 is a target for covalent EGFR kinase inhibitors and the use of third-generation EGFR-TKI such as AZD9291 has led to novel acquired resistance mechanisms including a newly acquired cysteine to serine mutation (C797S)^13,15^. This begs the question whether redox-dependent modifications of C797, or acquisition of C797S mutations, may have consequences for EGFR internalization and recycling dynamics, and/or nuclear EGFR functions, which has to our knowledge not been addressed to date. It is, however, also important to recognize that we cannot rule out the involvement of other cysteines within EGFR in such regulation. Indeed, detectable sulfenylation was observed in C797S EGFR mutants^26^, which likely involves alternative cysteines within EGFR. Intriguingly, recent work has identified several cysteines within the C-terminal autophosphorylation tail of EGFR as targets for palmitoylation, thereby regulating binding to Grb2 as well as receptor turnover^58^. Mutations of these cysteines were found to enhance EGFR signaling and promote cell migration and anchorage-independent growth, and redox-dependent modifications of these cysteines could conceivably also affect these processes. Hence, additional studies with various EGFR cysteine mutants will be required to definitively address this possibility. As alluded to earlier, another limitation of our studies is that we only addressed redox-dependent modifications within EGFR, whereas similar redox modifications in other proteins (e.g. involved in cell metabolism, subcellular trafficking, nuclear import, etc.) could also be altered. Indeed, recent proteomic studies indicate the contribution of DUOX1 to redox-dependent regulation of many diverse proteins in addition to EGFR^54^, and a more complete understanding of the consequences of DUOX1 silencing in lung cancer would also require a more thorough analysis of redox modifications of these other targets. Finally, in addition to trafficking to the nucleus, EGFR can also translocate to mitochondria and regulate mitochondrial function, which has potential consequences for cancer biology and metastatic potential^59,60^. It would be interesting to determine whether altered redox regulation related to DUOX1 status also impacts on mitochondrial EGFR trafficking.

In conclusion, our studies highlight the importance of disturbed redox-dependent mechanisms in controlling diverse EGFR functions in lung cancers in association with loss of DUOX1, and introduce the novel concept that enhanced turnover of EGFR cysteine oxidation in lung cancers, mediated by GSTP1-mediated *S*-glutathionylation and subsequent reduction, is responsible for enhanced nuclear EGFR localization and increased tumorigenic properties by kinase-independent functions. A more complete understanding of dynamic redox-dependent mechanisms involved in regulating diverse functional aspects of EGFR is likely to contribute to development of improved anticancer strategies.

## Methods

Detailed experimental procedures can be found in the Supplemental Information.

### Cell culture and experimentation

NCI-H292 cells, a human pulmonary mucoepidermoid carcinoma cell line (ATCC), were propagated in RPMI 1640 medium with 10% FBS/5% penicillin-streptomycin. DUOX1-deficient H292 cells (H292-shDUOX1) and corresponding control cells (H292-shCTL) were generated and maintained as described previously^9^. Alveolar adenocarcinoma A549 cells (ATCC), as well as A549 cells transfected with *DUOX1* cDNA (A549-pDUOX1) or empty vector (A549-pCTL) as described previously^9^, were maintained in DMEM-F12 media supplemented with neomycin in case of stably transfected cell lines. NCI-H187 human lung retinoblastoma cells (ATCC), similarly transfected with *DUOX1* cDNA (H187-pDUOX1) or empty vector controls (H187-pCTL) as previously described^9^, and H460 human lung carcinoma cells (ATCC) were maintained in RPMI 1640 medium with 10% FBS/5% penicillin-streptomycin. Overexpression or silencing of DUOX1 mRNA and protein in these various cell lines was characterized in detail^9^. Cells were cultured overnight in serum-free media prior to appropriate stimulation and analyses. The importance of GSPT1 was determined by pre-incubation with targeted siRNA (Dharmacon SmartPool siRNA # L-011179–00–0005, GE, Lafayette, CO) or non-targeting control siRNA (Dharmacon, GE, Lafayette, CO),

### Analysis of nuclear EGFR

Unstimulated or EGF-stimulated cells (100 ng/mL) (Millipore, MA, US) were PFA-fixed and permeabilized for analysis of EGFR (anti-EGFR; 1:100; Cell Signaling) and DAPI counterstaining, and imaged on a Zeiss LSM 510 META laser scanning confocal microscope (Zeiss, Jena, Germany). Cell lysates or nuclear extracts (prepared using a Subcellular Protein Fractionation Kit; #78840; Thermo Scientific, Waltham, MA, USA) were analyzed by Western blot for EGFR (1:1000; Cell Signaling) and phosphorylated forms (pEGFR-1068; 1:1000; Cell Signaling, or pEGFR-1101; 1:500; Abcam, Cambridge, MA). Gene expression associated with nEGFR was determined by RT-qPCR.

### Functional analyses

Cell proliferation and resistance to EGFR blocking were determined using ATP TiterGlo assay reagent (Promega, WI, USA). Cell migratory capacity was assessed in a scratch wound assay, and analysis of wound closure by brightfield imaging and analysis using NIH Image J.

### Analysis of EGFR cysteine oxidation

Cysteine oxidation status of EGFR was determined by derivatization with sulfenic acid probe DCP-bio1 (Kerafast, Boston, MA), cell pre-loading with biotinylated glutathione ethyl ester (BioGEE), or derivatization with EZ-link Iodoacetyl-LC-biotin (Pierce, Rockford, Ill), to assess sulfenylation, *S*-glutathionylation, and cysteine thiol status, respectively. Biotin-tagged proteins were purified with NeutrAvidin-agarose beads and analyzed by Western blotting with α-EGFR.

### Data presentation and statistical analyses

Quantitative data are represented as mean ± s.d. and were analyzed by one-way ANOVA or two-tailed Student’s t-test for statistical differences, which were considered significant when p < 0.05.

## Supplementary information

Supplementary Information

## Data Availability

All data generated or analyzed during the study are included in this published article (and its Supplementary Information files).

## References

[1] Reck M, Rabe KF (2017). Precision Diagnosis and Treatment for Advanced Non-Small-Cell Lung Cancer. N Engl J Med.

[2] Herbst RS, Morgensztern D, Boshoff C (2018). The biology and management of non-small cell lung cancer. Nature.

[3] Gridelli C (2015). Non-small-cell lung cancer. Nat Rev Dis Primers.

[4] Trachootham D, Alexandre J, Huang P (2009). Targeting cancer cells by ROS-mediated mechanisms: a radical therapeutic approach?. Nat Rev Drug Discov.

[5] Little AC (2017). Paradoxical roles of dual oxidases in cancer biology. Free Radic Biol Med.

[6] Luxen S, Belinsky SA, Knaus UG (2008). Silencing of DUOX NADPH oxidases by promoter hypermethylation in lung cancer. Cancer Res.

[7] Juhasz A (2009). Expression of NADPH oxidase homologues and accessory genes in human cancer cell lines, tumours and adjacent normal tissues. Free Radic Res.

[8] van der Vliet A, Danyal K, Heppner DE (2018). Dual oxidase: a novel therapeutic target in allergic disease. Br J Pharmacol.

[9] Little AC (2016). DUOX1 silencing in lung cancer promotes EMT, cancer stem cell characteristics and invasive properties. Oncogenesis.

[10] Camidge DR, Pao W, Sequist LV (2014). Acquired resistance to TKIs in solid tumours: learning from lung cancer. Nat Rev Clin Oncol.

[11] Wheeler DL, Dunn EF, Harari PM (2010). Understanding resistance to EGFR inhibitors-impact on future treatment strategies. Nat Rev Clin Oncol.

[12] Chong CR, Janne PA (2013). The quest to overcome resistance to EGFR-targeted therapies in cancer. Nat Med.

[13] Jia Y (2016). Overcoming EGFR(T790M) and EGFR(C797S) resistance with mutant-selective allosteric inhibitors. Nature.

[14] Patel H, Pawara R, Ansari A, Surana S (2017). Recent updates on third generation EGFR inhibitors and emergence of fourth generation EGFR inhibitors to combat C797S resistance. Eur J Med Chem.

[15] Thress KS (2015). Acquired EGFR C797S mutation mediates resistance to AZD9291 in non-small cell lung cancer harboring EGFR T790M. Nat Med.

[16] Madshus IH, Stang E (2009). Internalization and intracellular sorting of the EGF receptor: a model for understanding the mechanisms of receptor trafficking. J Cell Sci.

[17] Goh LK, Sorkin A (2013). Endocytosis of receptor tyrosine kinases. Cold Spring Harb Perspect Biol.

[18] Lo HW (2006). Nuclear-cytoplasmic transport of EGFR involves receptor endocytosis, importin beta1 and CRM1. J Cell Biochem.

[19] Wang YN (2010). The translocon Sec. 61beta localized in the inner nuclear membrane transports membrane-embedded EGF receptor to the nucleus. J Biol Chem.

[20] Biscardi JS (1999). c-Src-mediated phosphorylation of the epidermal growth factor receptor on Tyr845 and Tyr1101 is associated with modulation of receptor function. J Biol Chem.

[21] Iida M, Brand TM, Campbell DA, Li C, Wheeler DL (2013). Yes and Lyn play a role in nuclear translocation of the epidermal growth factor receptor. Oncogene.

[22] Brand TM (2013). Nuclear EGFR as a molecular target in cancer. Radiother Oncol.

[23] Traynor AM (2013). Nuclear EGFR protein expression predicts poor survival in early stage non-small cell lung cancer. Lung Cancer.

[24] Li C, Iida M, Dunn EF, Ghia AJ, Wheeler DL (2009). Nuclear EGFR contributes to acquired resistance to cetuximab. Oncogene.

[25] Paulsen CE (2012). Peroxide-dependent sulfenylation of the EGFR catalytic site enhances kinase activity. Nat Chem Biol.

[26] Truong TH (2016). Molecular Basis for Redox Activation of Epidermal Growth Factor Receptor Kinase. Cell Chem Biol.

[27] Heppner DE (2016). The NADPH Oxidases DUOX1 and NOX2 Play Distinct Roles in Redox Regulation of Epidermal Growth Factor Receptor Signaling. J Biol Chem.

[28] Hristova M (2016). Airway epithelial dual oxidase 1 mediates allergen-induced IL-33 secretion and activation of type 2 immune responses. J Allergy Clin Immunol.

[29] Sham D, Wesley UV, Hristova M, van der Vliet A (2013). ATP-mediated transactivation of the epidermal growth factor receptor in airway epithelial cells involves DUOX1-dependent oxidation of Src and ADAM17. PLoS One.

[30] Janssen-Heininger YM (2008). Redox-based regulation of signal transduction: principles, pitfalls, and promises. Free Radic Biol Med.

[31] Okamura T (2015). Phosphorylation of Glutathione S-Transferase P1 (GSTP1) by Epidermal Growth Factor Receptor (EGFR) Promotes Formation of the GSTP1-c-Jun N-terminal kinase (JNK) Complex and Suppresses JNK Downstream Signaling and Apoptosis in Brain Tumor Cells. J Biol Chem.

[32] Ali-Osman F, Brunner JM, Kutluk TM, Hess K (1997). Prognostic significance of glutathione S-transferase pi expression and subcellular localization in human gliomas. Clin Cancer Res.

[33] Louie SM (2016). GSTP1 Is a Driver of Triple-Negative Breast Cancer Cell Metabolism and Pathogenicity. Cell Chem Biol.

[34] Casaletto JB, McClatchey AI (2012). Spatial regulation of receptor tyrosine kinases in development and cancer. Nat Rev Cancer.

[35] Habibovic A (2016). DUOX1 mediates persistent epithelial EGFR activation, mucous cell metaplasia, and airway remodeling during allergic asthma. JCI Insight.

[36] Heppner DE, van der Vliet A (2016). Redox-dependent regulation of epidermal growth factor receptor signaling. Redox Biol.

[37] Wani R, Nagata A, Murray BW (2014). Protein redox chemistry: post-translational cysteine modifications that regulate signal transduction and drug pharmacology. Front Pharmacol.

[38] Bollu LR (2014). Involvement of de novo synthesized palmitate and mitochondrial EGFR in EGF induced mitochondrial fusion of cancer cells. Cell Cycle.

[39] Anathy V (2012). Oxidative processing of latent Fas in the endoplasmic reticulum controls the strength of apoptosis. Mol Cell Biol.

[40] Wang SB (2018). Protein S-Nitrosylation Controls Glycogen Synthase Kinase 3beta Function Independent of Its Phosphorylation State. Circ Res.

[41] Kitamura H, Motohashi H (2018). NRF2 addiction in cancer cells. Cancer Sci.

[42] Tew KD, Townsend DM (2011). Regulatory functions of glutathione S-transferase P1-1 unrelated to detoxification. Drug Metab Rev.

[43] Powis G, Kirkpatrick DL (2007). Thioredoxin signaling as a target for cancer therapy. Curr Opin Pharmacol.

[44] Espinosa, B. & Arner, E. S. J. Thioredoxin related protein of 14 kDa as a modulator of redox signaling pathways. *Br J Pharmacol* (2018). 10.1111/bph.14479 PMC6346142 30129655

[45] Sirokmany G (2016). Epidermal growth factor-induced hydrogen peroxide production is mediated by dual oxidase 1. Free Radic Biol Med.

[46] Sigismund S (2008). Clathrin-mediated internalization is essential for sustained EGFR signaling but dispensable for degradation. Dev Cell.

[47] De Angelis Campos AC (2011). Epidermal growth factor receptors destined for the nucleus are internalized via a clathrin-dependent pathway. Biochem Biophys Res Commun.

[48] Mutch LJ, Howden JD, Jenner EP, Poulter NS, Rappoport JZ (2014). Polarised clathrin-mediated endocytosis of EGFR during chemotactic invasion. Traffic.

[49] Delos Santos RC (2017). Selective regulation of clathrin-mediated epidermal growth factor receptor signaling and endocytosis by phospholipase C and calcium. Mol Biol Cell.

[50] Caldieri G (2017). Reticulon 3-dependent ER-PM contact sites control EGFR nonclathrin endocytosis. Science.

[51] Myromslien FD (2006). Both clathrin-positive and -negative coats are involved in endosomal sorting of the EGF receptor. Exp Cell Res.

[52] van der Vliet A (2008). NADPH oxidases in lung biology and pathology: host defense enzymes, and more. Free Radic Biol Med.

[53] De Deken X, Corvilain B, Dumont JE, Miot F (2014). Roles of DUOX-mediated hydrogen peroxide in metabolism, host defense, and signaling. Antioxid Redox Signal.

[54] Hristova M (2014). Identification of DUOX1-dependent redox signaling through protein S-glutathionylation in airway epithelial cells. Redox Biol.

[55] Park S (2018). Inactivation of the PtdIns(4)P phosphatase Sac1 at the Golgi by H2O2 produced via Ca(2+)-dependent Duox in EGF-stimulated cells. Free Radic Biol Med.

[56] Henmi Y (2016). PtdIns4KIIalpha generates endosomal PtdIns(4)P and is required for receptor sorting at early endosomes. Mol Biol Cell.

[57] Kong LL (2017). Structural pharmacological studies on EGFR T790M/C797S. Biochem Biophys Res Commun.

[58] Runkle KB (2016). Inhibition of DHHC20-Mediated EGFR Palmitoylation Creates a Dependence on EGFR Signaling. Mol Cell.

[59] Demory ML (2009). Epidermal growth factor receptor translocation to the mitochondria: regulation and effect. J Biol Chem.

[60] Che TF (2015). Mitochondrial translocation of EGFR regulates mitochondria dynamics and promotes metastasis in NSCLC. Oncotarget.

