# Supplementary material for: Dysregulated Redox Regulation Contributes to Nuclear EGFR Localization and Pathogenicity in Lung Cancer
Source: Sci Rep. 2019 Mar 19;9:4844. doi: 10.1038/s41598-019-41395-8 (PMC6425021; doi:10.1038/s41598-019-41395-8)

SUPPLEMENTAL INFORMATION

Dysregulated Redox Regulation Contributes to Nuclear EGFR Localization and Pathogenicity in Lung Cancer

Andrew C. Little[1,2], Milena Hristova[1], Loes van Lith[1], Caspar Schiffers[1], Christopher M. Dustin[1], Aida Habibovic[1], Karamatullah Danyal[1], David E. Heppner[1,3], Miao-Chong Lin[1], Jos van der Velden[1], Yvonne M. Janssen-Heininger[1], and Albert van der Vliet[1,*]

[1]Department of Pathology and Laboratory Medicine, Robert Larner, M.D. College of Medicine, University of Vermont, Burlington, VT 05405, USA; [2]: Current address: Rogel Cancer Center, Department of Internal Medicine Hematology-Oncology, University of Michigan, MI, USA; [3]: Current address: Department of Cancer Biology, Dana-Farber Cancer Institute, Boston, MA 02215, U.S.A. Department of Biological Chemistry and Molecular Pharmacology, Harvard Medical School, Boston, MA 02115, U.S.A.

Experimental Procedures

Cell lines and transfections - NCI-H292 cells, a human pulmonary mucoepidermoid carcinoma cell line (ATCC), was propagated in RPMI 1640 medium with 10% FBS/5% penicillin-streptomycin. DUOX1-deficient H292 cells (H292-shDUOX1) and corresponding control cells (H292-shCTL) were generated and maintained as described previously[1]. Alveolar adenocarcinoma A549 cells (ATCC), as well as A549 cells transfected with *DUOX1* cDNA (A549-pDUOX1) or empty vector (A549-pCTL) as described previously[1], were maintained in DMEM-F12 media supplemented with neomycin in case of stably transfected cell lines. NCI-H187 human lung retinoblastoma cells (ATCC), similarly transfected with *DUOX1* cDNA (H187-pDUOX1) or empty vector controls (H187-pCTL) as previously described [1], and H460 human lung carcinoma cells (ATCC) were maintained in RPMI 1640 medium with 10% FBS/5% penicillin-streptomycin. Overexpression or silencing or DUOX1 mRNA and protein in these various cell lines was characterized in detail[1]. Cells were cultured overnight in serum-free media prior to experimentation and analyses.

RNA extraction and RT-PCR – Total RNA was extracted with GeneJET RNA purification kit (Thermo Scientific, Waltham, MA, USA). RNA extracts were reverse-transcribed and real-time PCR was performed using SYBR green qPCR assays. All qPCR primers (Supplemental Table 1) were purchased from Sigma-Aldrich.

Immunofluorescence analysis – Cells of interest were seeded in 8-well glass chamber slides, treated with EGF (100ng/mL) (Millipore, MA, US), and fixed with 4% PFA, and permeabilized with 0.2% Triton X-100 in 1% bovine serum albumin (BSA) in PBS for 15 min. Slides were

washed 2X with 1% BSA/PBS solution, blocked with 10% goat serum for 1 hr, and then subjected to staining with antibody against EGFR (1:100; #42678S; Cell Signaling) overnight at 4°C. Nuclei were counterstained with DAPI (10 µg/mL in 1% BSA/PBS). Cells were washed 2X with 1% BSA/PBS, mounted with glass coverslip, and imaged on a Zeiss LSM 510 META laser scanning confocal microscope (Zeiss, Jena, Germany). Brightfield images were taken using a Zeiss Interskop phase contrast microscope interfaced with a digital camera (Zeiss, Jena, Germany).

Analysis of nuclear EGFR by immune-electron microscopy – Cells of interest were cultured to ~70% confluence on glass chamber slides and provided to the University of Vermont Microscopy and Imaging Core Facility for further processing. For immunoEM staining, cells were fixed with 4% paraformaldehyde for 30 minutes followed by 3X wash with 1X PBS. Cells were then subjected to heat induced antigen retrieval in a decloaking chamber for 95°C for 15 minutes, followed by 90°C for 1 minute. Samples were cooled for 15 minutes at room temperature and then preconditioned on filtered drops of PBS/1%BSA/0.1% Tween 20 for 30 minutes at room temperature. Primary antibody (EGFR XP; Cell Signaling #4267S; 1:1000) was incubated o/n at 4°C. Samples were washed 3X with PBS/1% BSA. Samples were subject to secondary silver particle incubation (pAg_{10}), diluted in PBS (O.D. 0.06 at 525nm) for 60 minutes at room temperature. Samples were washed 2X with PBS/1%BSA and finished with a DI water wash 3X. Samples were imaged on a JEOL 1400 Transmission Electron Microscope (JEOL USA, Inc. Peabody, MA, USA) at 80kV, 8kx.

Cell lysis, subcellular fractionation, and Western blot analyses – Cells were cultured and subjected to appropriate treatments in 24-well culture plates, and lysed using 100 µL of 1X western

solubilization lysis buffer (1% Triton, 50 mM HEPES, 250 mM NaCl, 10% glycerol, 1.5 mM MgCl, 1 mM PMSF, 1 mM EGTA, 2 mM Na_3VO_4, 10 μg/mL aprotinin, 10 μg/mL leupeptin; pH 7.4) per well. Alternatively, subcellular protein isolation was performed with the Subcellular Protein Fractionation Kit (#78840; Thermo Scientific, Waltham, MA, USA), in accordance with manufacturer's protocol, resulting in nuclear and cytosol+membrane extracts. For Western blot analyses, equal amounts of protein (20-25 μg; determined using BCA protein assay) were separated on Novex 10% or 12% Tris-Glycine gels (Life Technologies, Grand Island, NY), transferred to nitrocellulose membranes, and blotted using antibodies against: EGFR (1:1000; Cell Signaling), pEGFR-1068 (1:1000; Cell Signaling), pEGFR-1101 (1:500; Abcam, Cambridge, MA), Histone H3 (1:2000; Cell Signaling), GAPDH (1:1000; Origene, Rockville, MD) and detected using enhanced chemiluminescence (Pierce, Rockfort, IL).

Assays of cell proliferation and migration – To analyze cell proliferation, cells were seeded at $1x10^4$ per well into a 96-well black wall-flat bottom fluorescence plates (Nunc International, Rochester, NY, USA) in serum-free medium, and media was supplemented with 100 ng/mL EGF up to 72 hrs, after which viable cells were analyzed with ATP Cell TiterGlo reagent (Promega, Madison, WI). To assess cell resistance against EGFR blocking, cells were seeded at $1x10^4$ per wells into a 96-well black wall-flat bottom fluorescence plates and cultured in complete media in the presence of 15 μg/mL of neutralizing EGFR mAb (#05-101, Millipore) or IgG isotype control Ab for 72 hrs, with fresh supplemented media provided daily. Cell viability was then determined using ATP TiterGlo assay reagent. Cell migratory capacity was assessed in a scratch wound assay, by applying a linear ~2 mm scratch in confluent cell monolayers in 24-well culture plates, removal of detached cells with PBS, and incubation in serum-free RPMI-1640 or DMEM-F12,

supplemented with either 100 ng/mL EGF or mock. Wound closure was monitored over 24 hrs, and imaged and analyzed using NIH Image J software to determine % wound closure.

Analysis of EGFR sulfenylation – To determine protein cysteine sulfenylation (-SOH), cells were lysed in Western solubilization buffer containing 1 mM of the sulfenic acid probe DCP-bio1 (Kerafast, Boston, MA), in the presence of 200 units/ml catalase (Worthington, Lakewood, NJ) and 10 mM *N*-ethylmaleimide (Sigma), and incubated for 1 h on ice, essentially as described previously [2,3]. Excess DCP-bio1 reagent was removed by 6 successive washes with 20 mM Tris-HCl (pH 7.4) on Amicon Ultra-0.5 Centrifugal Filter Devices (Millipore), and biotin-tagged proteins were collected with high capacity NeutrAvidin-agarose beads (50 μl of a 50/50 slurry; Pierce) and biotin-precipitated proteins were then analyzed by SDS-PAGE and western blotting against EGFR. Alternatively, EGFR was immunoprecipitated from DCP-Bio1-derivatized cell lysates (Cell Signaling) and analyzed by SDS-PAGE and streptavidin blotting.

Analysis of EGFR S-glutathionylation – To identify protein S-gluathionylation, cells were pre-loaded with biotinylated glutathione ethyl ester (BioGEE), prepared by reacting 0.5 M glutathione ethyl ester (Sigma) with 0.5 M EZ-link sulfo-NHS-biotin (Pierce) in 50 mM $NaHCO_3$ (pH 8.5) and added to cells at a final concentration of 250 μM, for 1 h prior to cell treatments. After cell lysis, excess BioGEE was removed using G25 columns (GE Healthcare) and biotin-tagged proteins (reflecting S-glutathionylated proteins) were isolated using NeutrAvidin-agarose beads, eluted with DTT and analyzed by Western blotting [4].

Analysis of EGFR thiol status – Cells were lysed in deoxygenated Western solubilization buffer containing 100 μmol/L EZ-link Iodoacetyl-LC-biotin (Pierce, Rockford, Ill) and 200 U/mL catalase under N_2 atmosphere (to avoid artificial cysteine oxidation) and incubated for 90 min at 37°C. Excess Iodoacetyl-LC-biotin reagent was removed using G25 columns, and biotin-tagged proteins were purified with NeutrAvidin-agarose beads and analyzed as described above.

Determination of Cellular Reactive Oxygen Species (ROS) – Cells were seeded in 96 well plates, pretreated with 5 μM of H_2DCFDA (#D399, Thermo) for 30 min at 37°C, and DCF fluorescence was monitored on a Bio-Tek Synergy HT fluorescent plate reader (Bio-Tek, Winooski, VT) in accordance with manufacturer's protocol. Alternatively, intracellular ROS levels were assessed with the H_2O_2 sensitive probe CellRox Deep Red Reagent (#C10422, Thermo) by pretreating cells seeded on 8-well chamber slides with 5 μM CellRox Deep Red for 30 min at 37°C, followed by paraformaldehyde fixation (4%) for 30 min at RT, and fluorescence imaging on a Zeiss LSM 510 META laser scanning confocal microscope (Zeiss). Quantification of CellRox signal was performed on ImageJ (NIH) from at least 2 independent experiments.

Suppression of GSTP1 by RNA interference – Cells were grown to ~70% confluence in 24-well plates and transfected with 0.1 μM siRNA targeted against GSTP1 (Dharmacon SmartPool siRNA # L-011179-00-0005, GE, Lafayette, CO), or 0.1 μM non-targeting control siRNA (Dharmacon, GE, Lafayette, CO), in either RPMI 1640 (H292 or H187 cells) or DMEM-F12 media (A549 cells) (Gibco, Life Technologies, Grand Island, NY), and incubated for 24 hrs at 37°C. Cells were then placed in complete media, and incubated for an additional 24 hrs prior to serum starvation and experimentation.

Data presentation and statistical analyses – Quantitative data are represented as mean ± s.d. and were analyzed by one-way ANOVA or Student’s t-test for statistical differences, which were considered significant when $p<0.05$.

SUPPLEMENTAL FIGURES

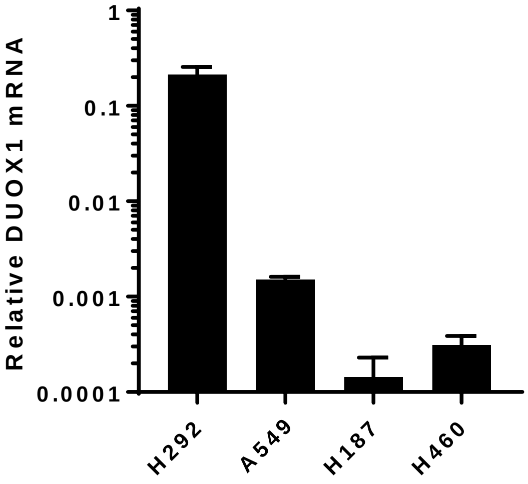

Figure S1: DUOX1 expression in cancer cell lines used in this study. Relative expression of DUOX1 in cancer cell lines was assessed by RT-PCR relative to GAPDH, and normalized to primary normal bronchial epithelial cells (NHBE, Lonza), using the $2^{-\Delta\Delta Ct}$ method. Mean ± S.E (n=3)

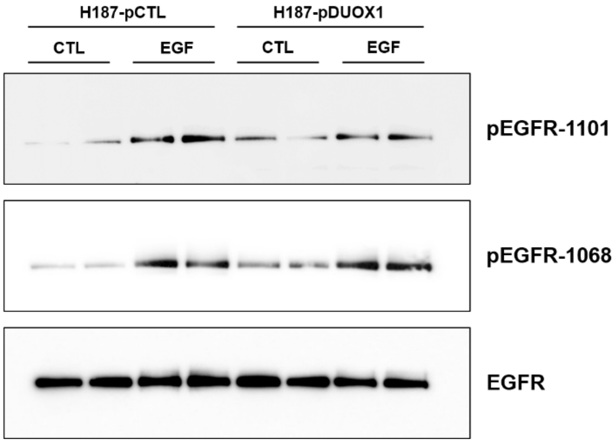

Figure S2. DUOX1 overexpression in H187 cells suppresses EGF-induced EGFR-Y1101 phosphorylation.

H187 empty vector cells (H187-pCTL) or H187 cells overexpressing DUOX1 (H187-pDUOX1), were treated with EGF ligand (100ng/mL) for 20 minutes and examined for EGFR phosphorylation events. Western blot image is representative of 2 separate experiments.

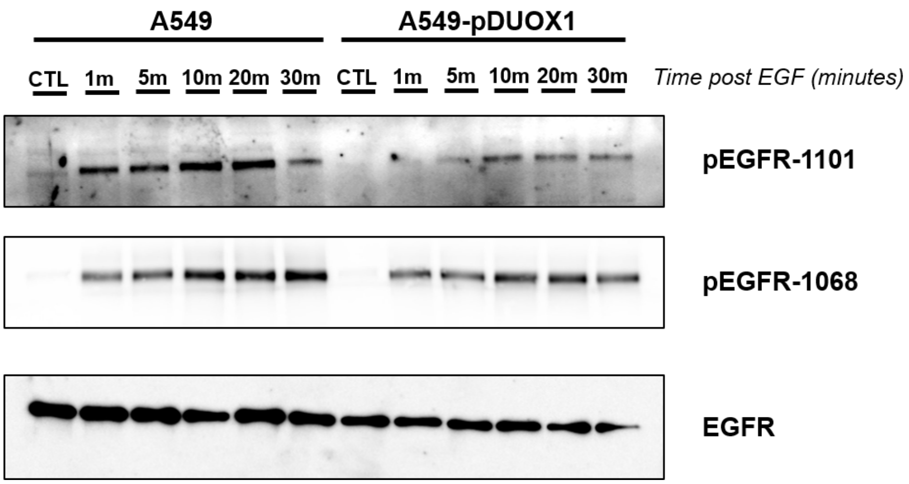

Figure S3. Time-dependent EGFR Y1101 phosphorylation in A549 cells and A549 cells overexpressing DUOX1.

Representative western blots display levels of EGFR-Y1101 phosphorylation following cell stimulation with 100 ng/mL EGF ligand for indicated time periods. Western blot images are representative of 2 separate experiments.

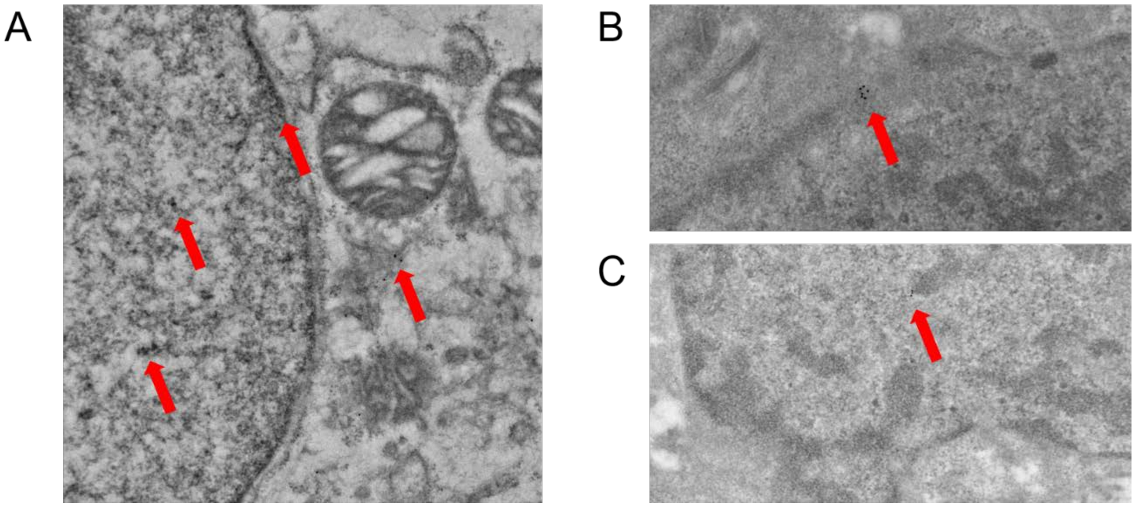

Figure S4. Detection of nEGFR using immuno-electron microscopy.

Results from immunoEM display EGFR can be detected (red arrows) in the perinuclear and internuclear spaces, as well as in the nuclear membrane in A549 cells (A) and H292 cells (B/C).

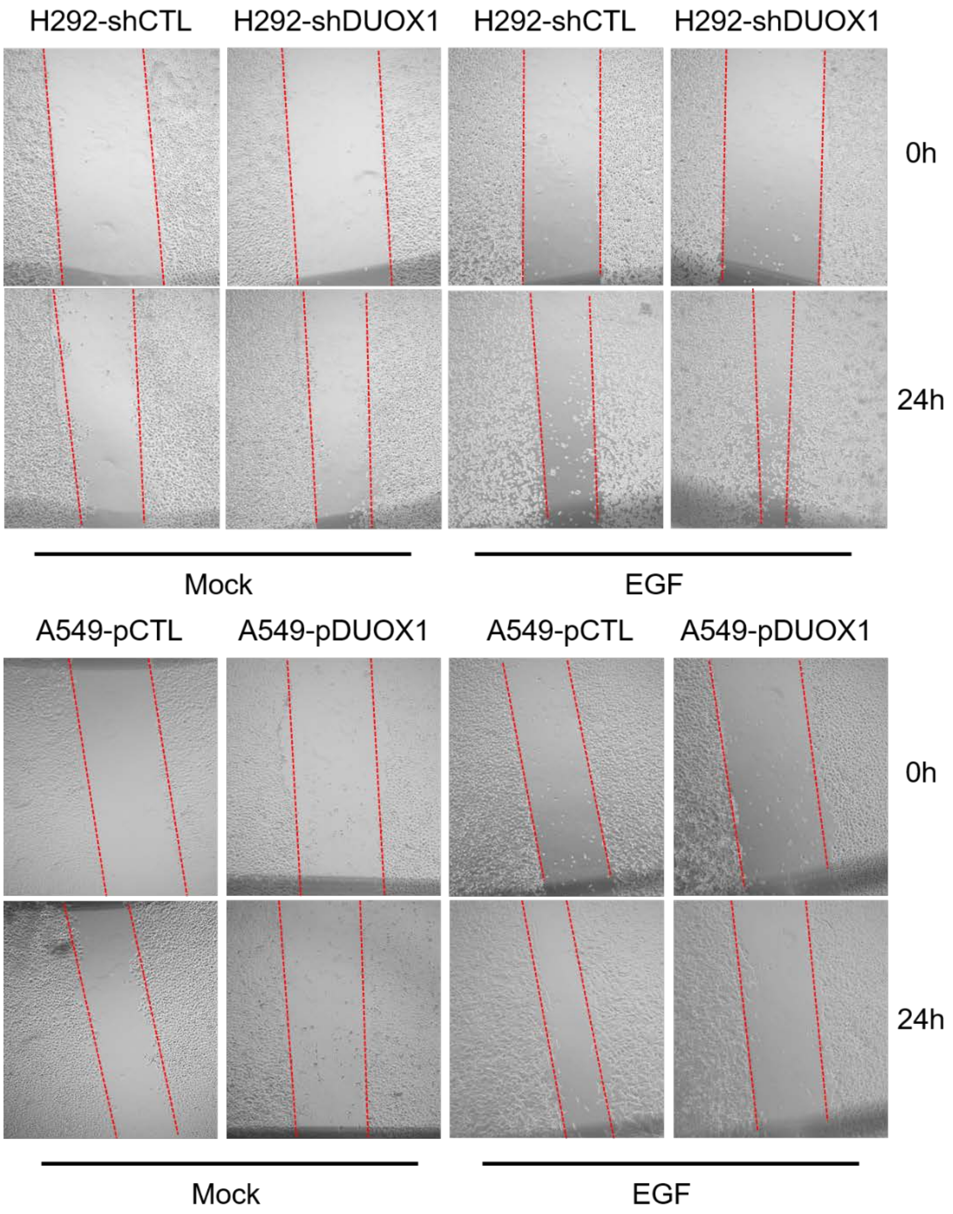

Figure S5. Representative images indicating EGF-stimulated wound closure depending on DUOX1 expression status cells.
H292 or A549 cells respond to EGF (100 ng/mL) with enhanced wound closure in a scratch assay, only when DUOX1 was absent (H292-shDUOX1; A549-pCTL) but not when DUOX1 was expressed (H292-shCTL; A549-pDUOX1). Images are representative of 2 separate experiments performed in quadruplicate.

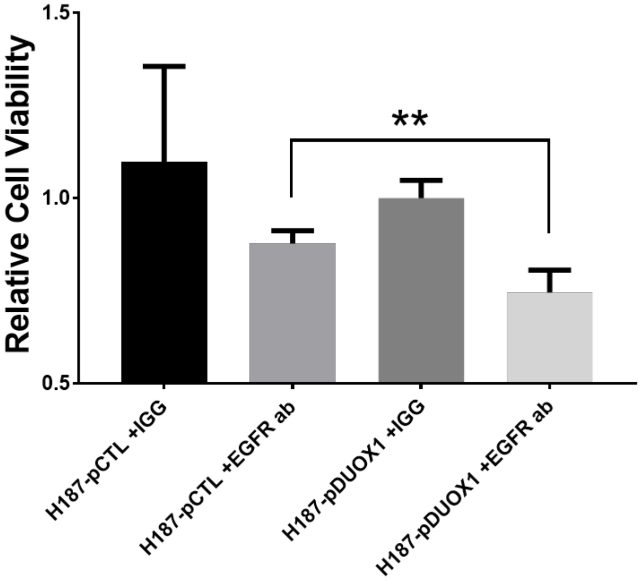

Figure S6. DUOX1-overexpressinon enhances sensitivity of H187 cells to EGFR mAb inhibition.

H187 cells overexpressing DUOX1 or corresponding controls were treated with 15 ng/mL EGFR blocking antibody for 72 hrs and cell viability determined with ATP-Cell-Titer-Glo reagent. Results are an average of 2 separate experiments, with 4 experimental replicates, **p<0.01; as determined by one-way ANOVA.

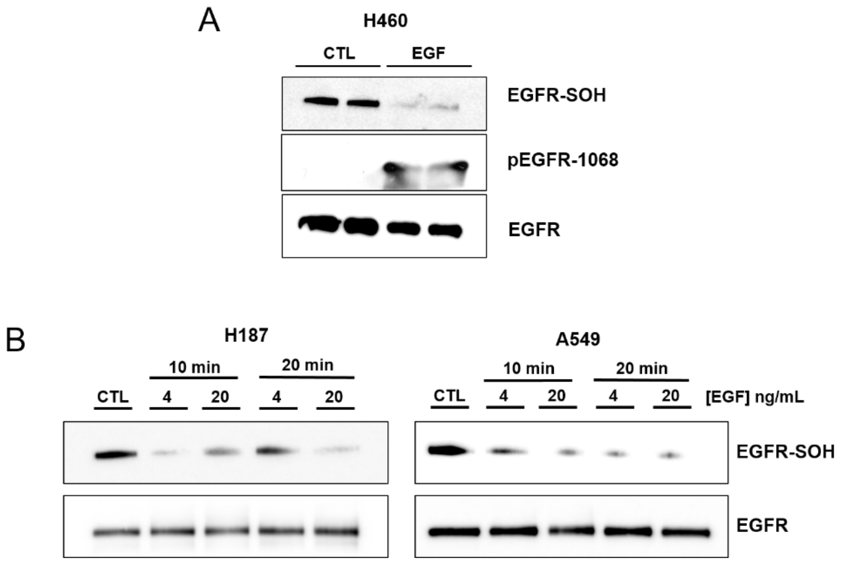

Figure S7. EGF-induced loss of EGFR sulfenylation in lung cancer cells.

(A) H460 lung cancer cells (which lack DUOX1) were stimulated with EGF (100 ng/mL; 20 min) and EGFR sulfenylation and phosphorylation was assessed. Western blot results are representative of 2 independent experiments.

(B) Stimulation of A549 or H187 cells lower EGF concentrations (4-20 ng/mL) also results in loss of EGFR sulfenylation. Western blot results are representative of 2 independent experiments.

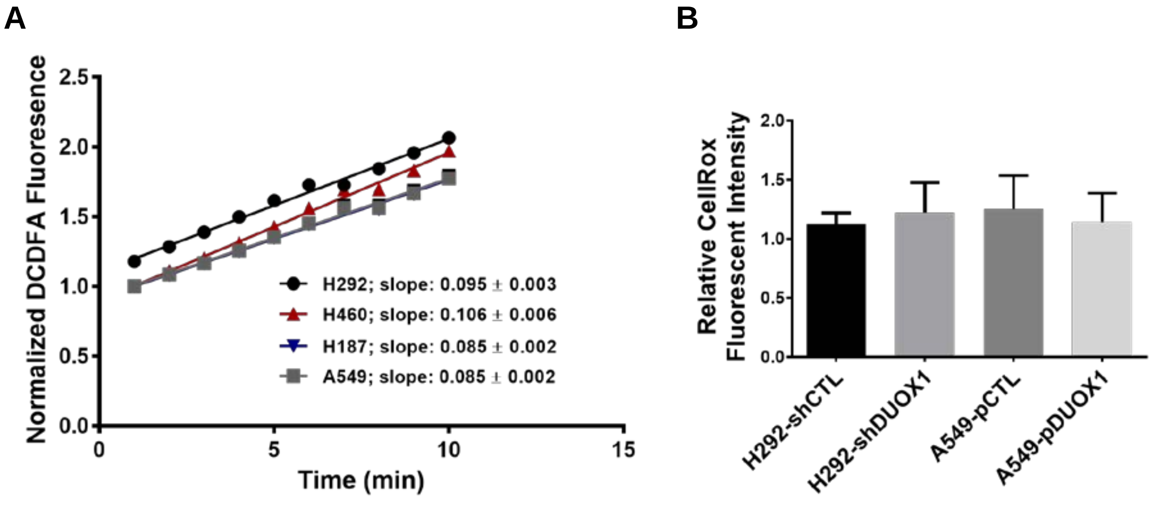

Figure S8. Basal rates of ROS accumulation are unchanged with respect to DUOX1 expression.

(A) Analysis of DCF fluorescence after cell loading with H_2DCF-DA, indicating similar rates of DCF accumulation in the different cell lines studied.

(B) Analysis of CellRox DeepRed fluorescence in H292 or A549 do not indicate significant changes after DUOX1 silencing or overexpression. Data are representative of at least 2 independent experiments.

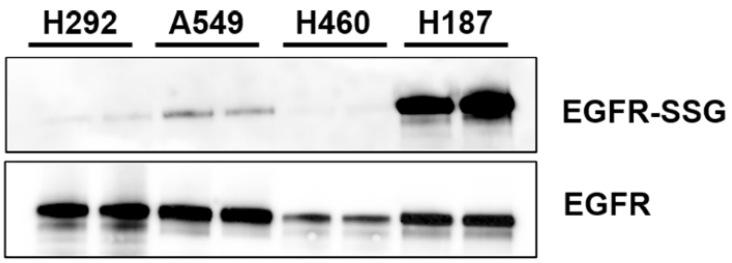

Figure S9. Basal S-glutathionylation of EGFR is enhanced in DUOX1-deficient lung cancer cells.

Cells were incubated with 250 µM biotin-GSH ester (BioGEE) for 1 hr prior to cell lysis and biotin tagged proteins were avidin-purified and analyzed with a-EGFR (top blot), compared to similar EGFR analysis in whole cell lysates (bottom).

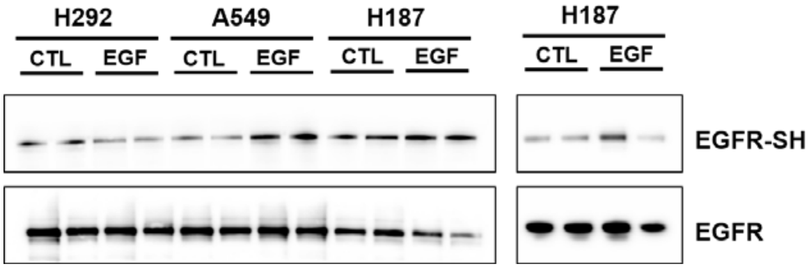

Figure S10. Effect of EGF stimulation on EGFR thiol status in various cancer cell lines.

Indicated cell lines were stimulated with EGF (100 ng/mL; 20 min) and lysed in the presence of iodoacetyl-LC-biotin to alkylate reduced cysteines. Avidin pull-downs (above) or whole lysates (below) were analyzed by Western blot for the presence of EGFR.

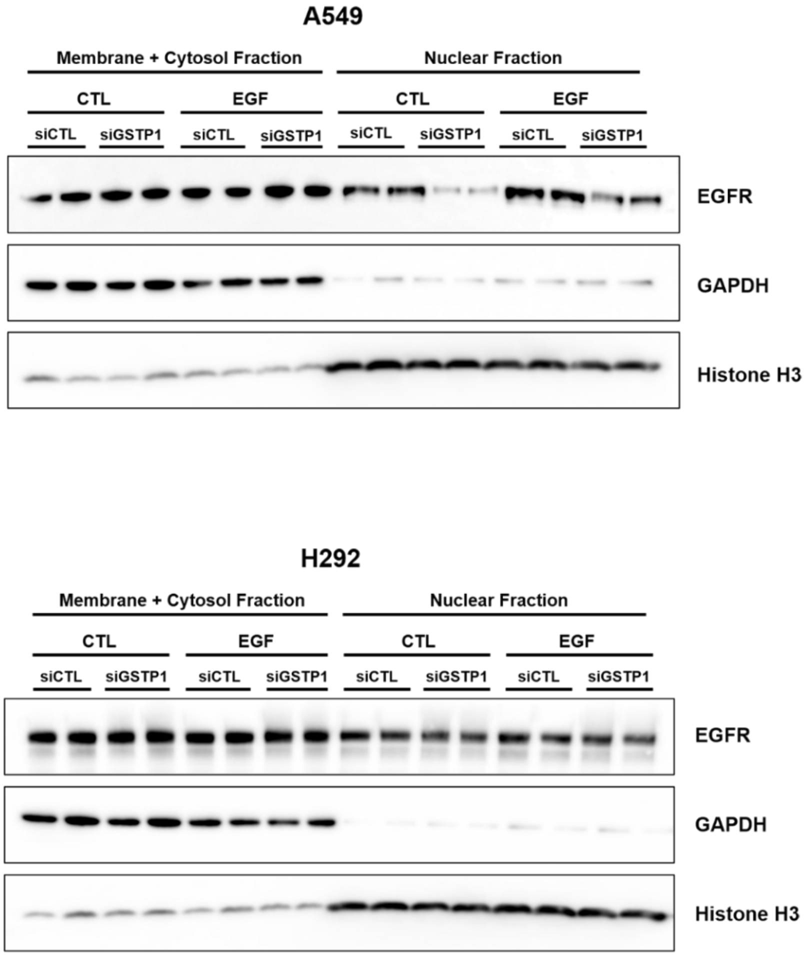

Figure S11. Effect of GSTP1 silencing on nuclear EGFR localization.

A549 cells (top) or H292 cells (bottom) were transfected with control or GSTP1-targeting siRNA and stimulated with EGF (100 ng/mL) after which cells were fractionated into membrane+cytosol and nuclear extracts, which were both analyzed for EGFR, GAPDH and the nuclear marker Histone H3. Representative Western blots from one of 2 separate experiments are shown.

Supplemental Table I. Quantitative Real-Time PCR primer sequences used in this study.

Gene Name	Primer	Sequence
DUOX1	Fwd	TTCACGCAGCTCTGTGTCAA
DUOX1	Rev	AGGGACAGATCATATCCTGGCT
AREG (amphiregulin)	Fwd	GTGTGGGGAAAAGTCCATGA
AREG	Rev	CTGGAAAGAGGACCGACTCA
EGF	Fwd	CAGGGAAGATGACCACCACT
EGF	Rev	TTCCCACCACTTCAGGTCTC
EREG (epiregulin)	Fwd	TCCATCTTCTACAGGCAGTCC
EREG	Rev	CACGGTCAAAGCCACATACTC
TGF alpha	Fwd	AGTGGTCTGAAGAGCCCAGA
TGF alpha	Rev	ATCTCTGGCAGTGCTGTCCT
AURKA	Fwd	AAACCATCAAATTGCCAAAA
AURKA	Rev	GTTCTAGATTGAGGGCAGCA
CCND1	Fwd	TTCAAATGTGTGCAGAAGGA
CCND1	Rev	GGGATGGTCTCCTTCATCTT
STAT1	Fwd	GCAAAACCTTGCAGAACAGA
STAT1	Rev	ATCAGGGCATTCTGGGTAAG
MYC	Fwd	CGACGAGACCTTCATCAAAA
MYC	Rev	TGCTGTCGTTGAGAGGGTAG
MYBL2	Fwd	TGGCTGAGAGTTTTGAATCC
MYBL2	Rev	TCCAGCAAGACTTCTTCACC
NOS2 (iNOS)	Fwd	GGAAGCCCCATAATTGTTCT
NOS2	Rev	ATTGGATGGGTTCATGTCAC
GAPDH	Fwd	GAAGGCTGGGGCTCATTTG
GAPDH	Rev	AGGCTGTTGTCATACTTCTCATGG

References

1. Little, A.C., *et al.* DUOX1 silencing in lung cancer promotes EMT, cancer stem cell characteristics and invasive properties. *Oncogenesis* **5**, e261 (2016).
2. Nelson, K.J., *et al.* Use of dimedone-based chemical probes for sulfenic acid detection methods to visualize and identify labeled proteins. *Methods Enzymol* **473**, 95-115 (2010).
3. Hristova, M., *et al.* Airway epithelial dual oxidase 1 mediates allergen-induced IL-33 secretion and activation of type 2 immune responses. *J Allergy Clin Immunol* **137**, 1545-1556 e1511 (2016).
4. Heppner, D.E., *et al.* The NADPH Oxidases DUOX1 and NOX2 Play Distinct Roles in Redox Regulation of Epidermal Growth Factor Receptor Signaling. *J Biol Chem* **291**, 23282-23293 (2016).

Uncropped western blots for Figures 1-4

Fig 1a

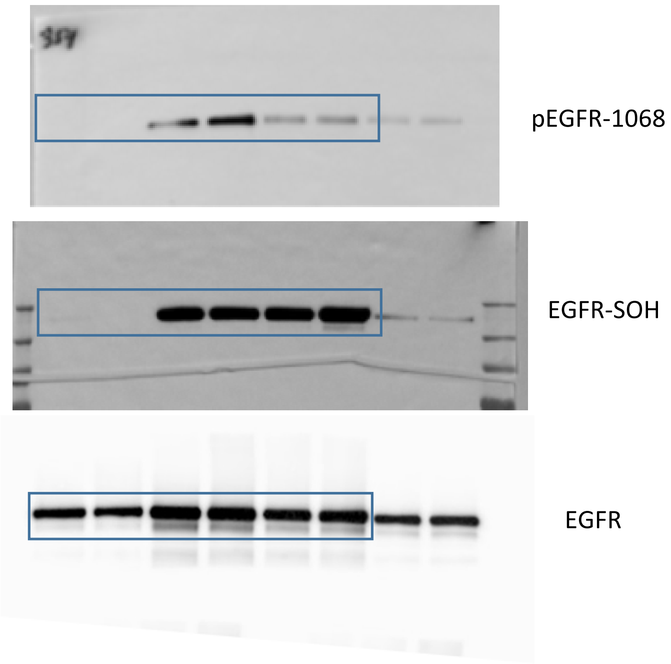

Fig 1e

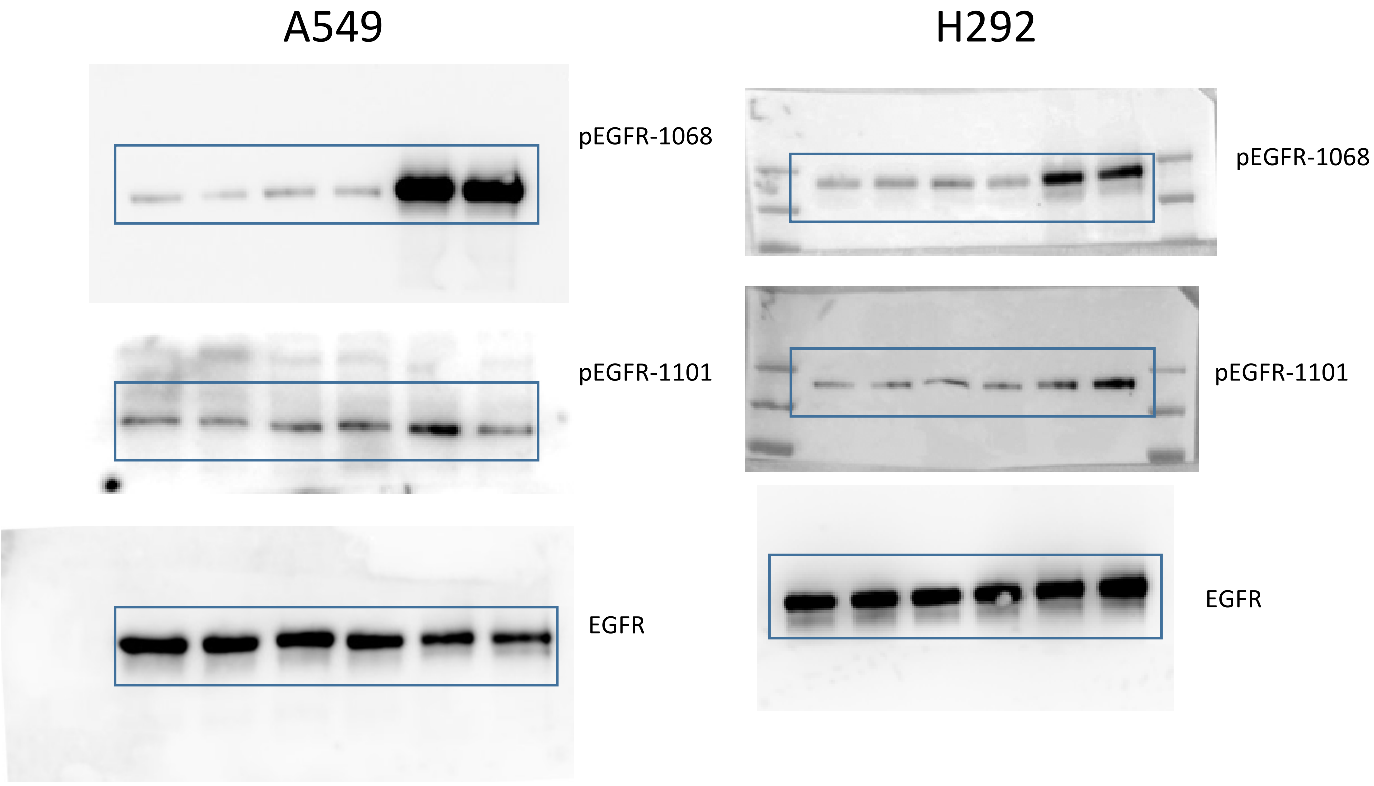

Fig 2a

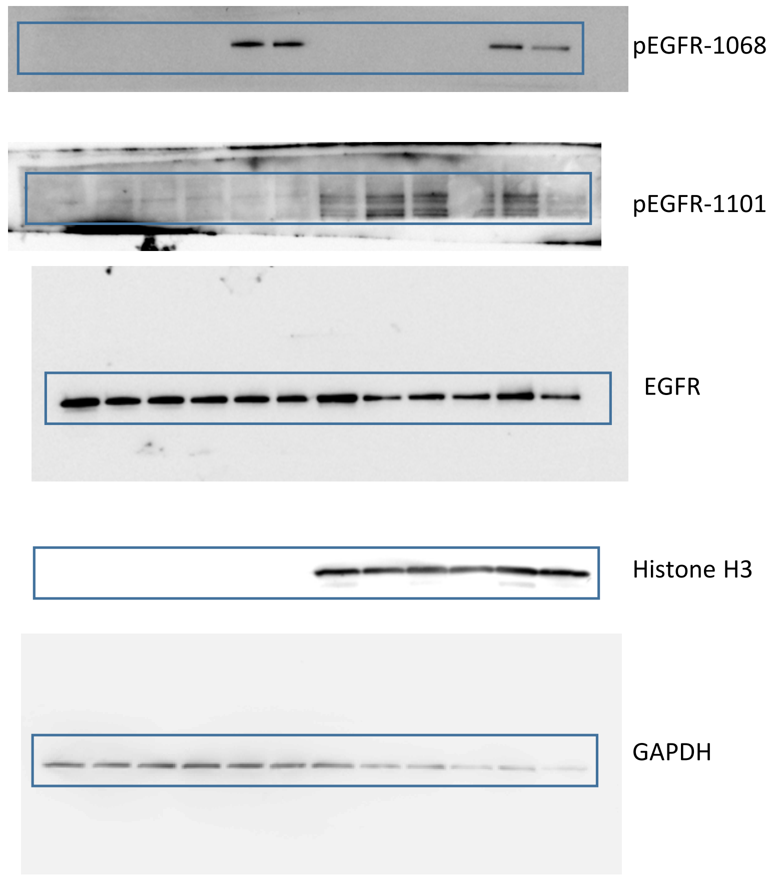
pEGFR-1068
pEGFR-1101
EGFR
Histone H3
GAPDH

Fig 2b

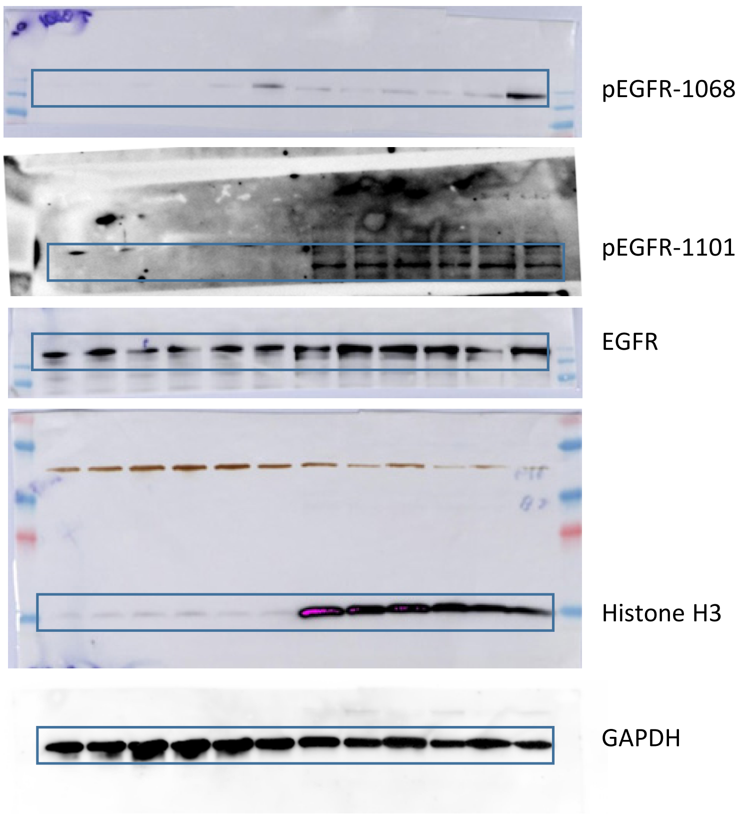
pEGFR-1068
pEGFR-1101
EGFR
Histone H3
GAPDH

Fig 3a

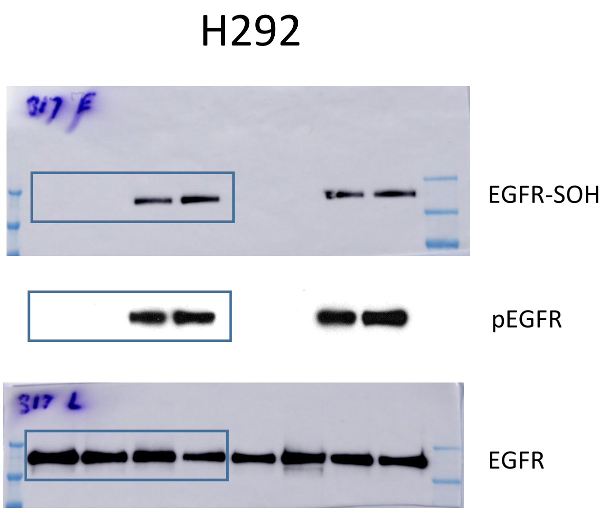
H292
EGFR-SOH
pEGFR
EGFR

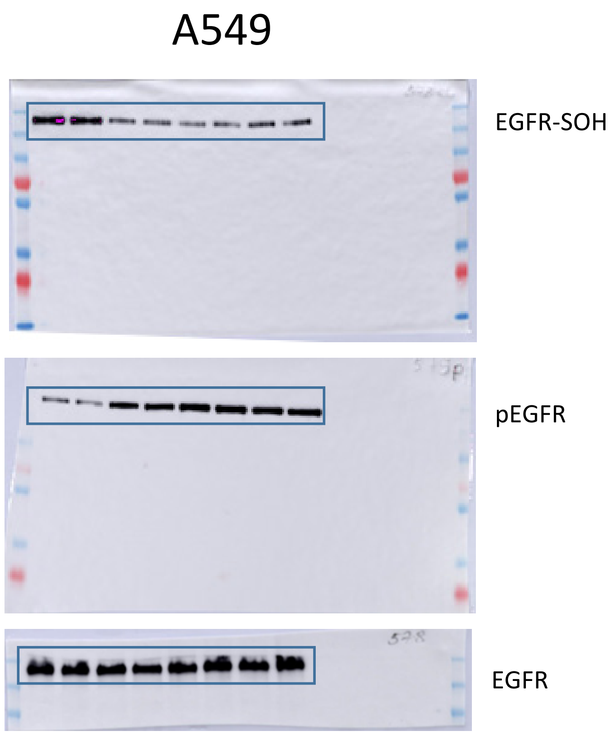
A549
EGFR-SOH
pEGFR
EGFR

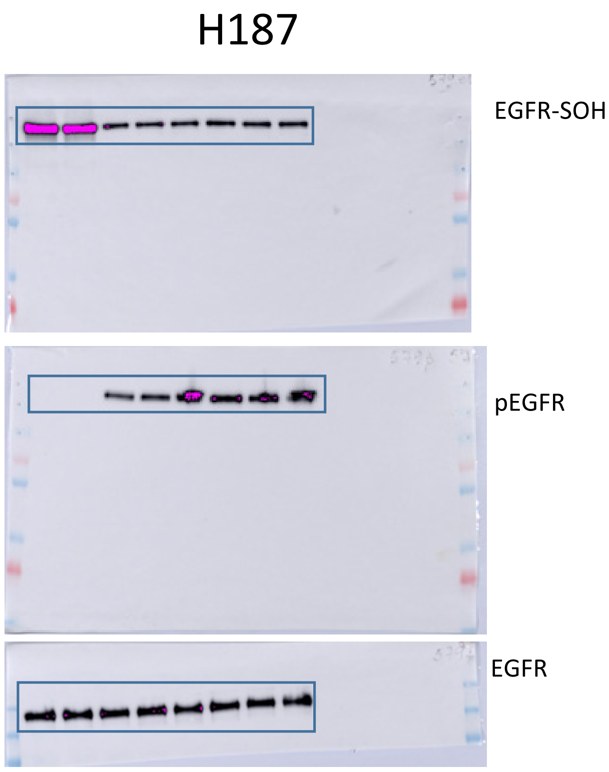
H187
EGFR-SOH
pEGFR
EGFR

Fig 3b

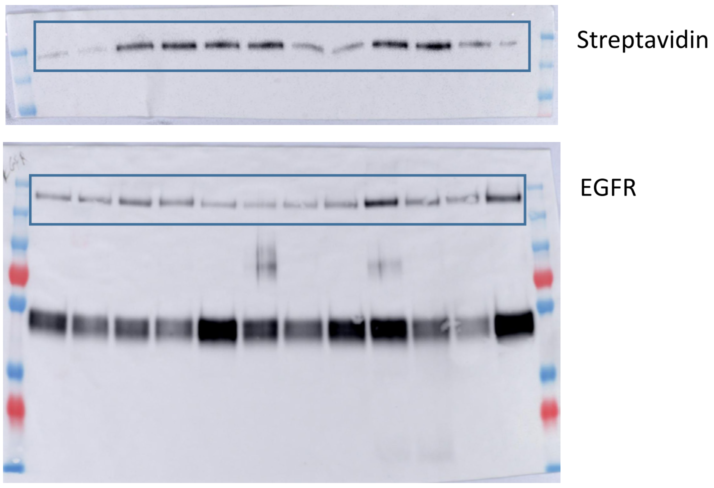
Streptavidin
EGFR

Fig 3c

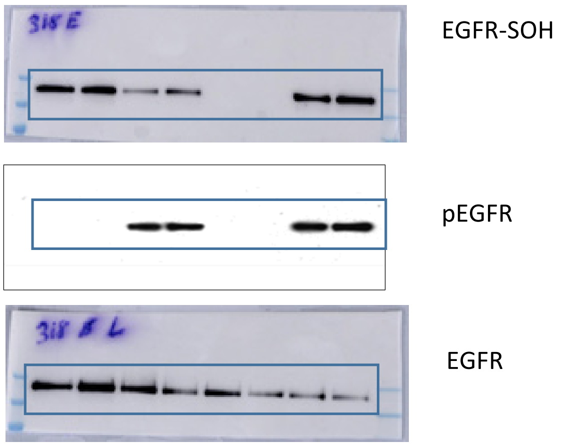

Fig 3d

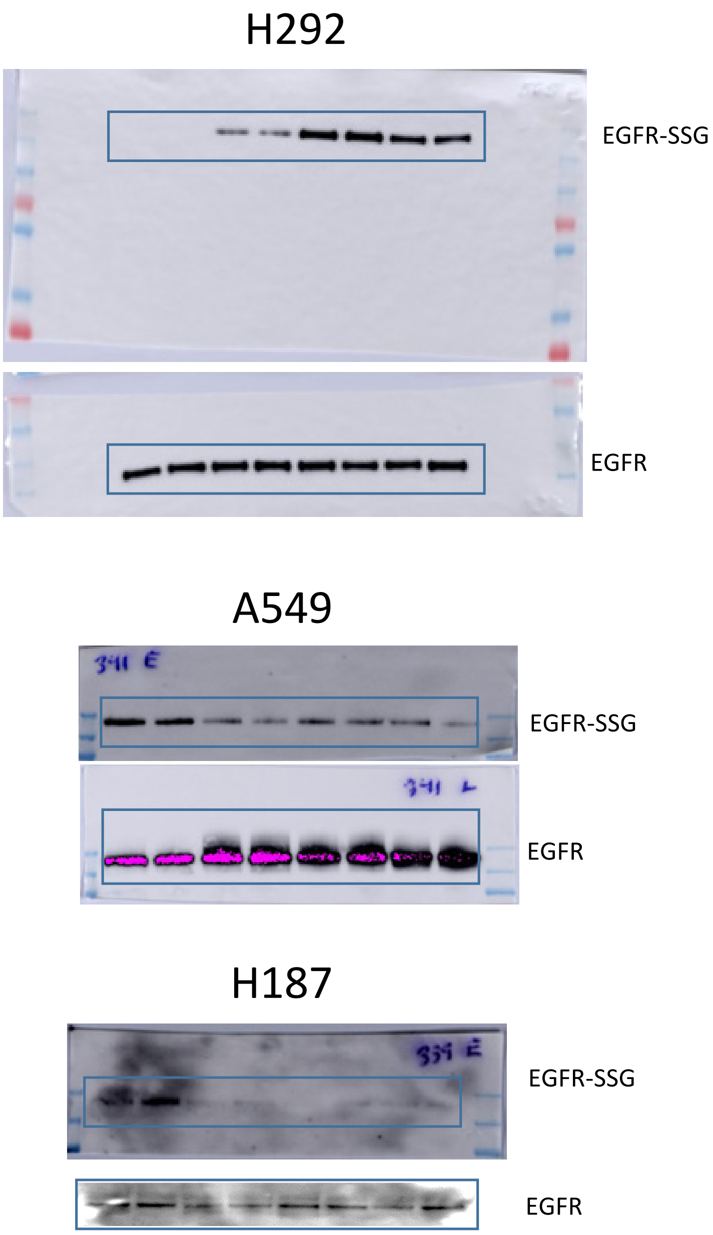

Fig 3e

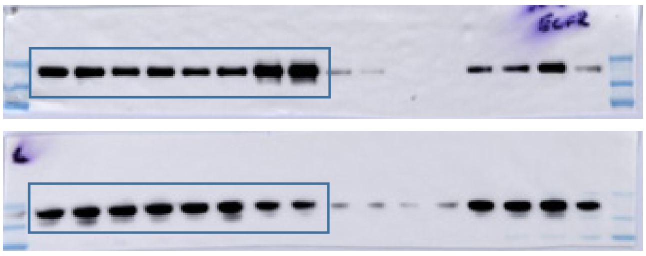

Fig 4a

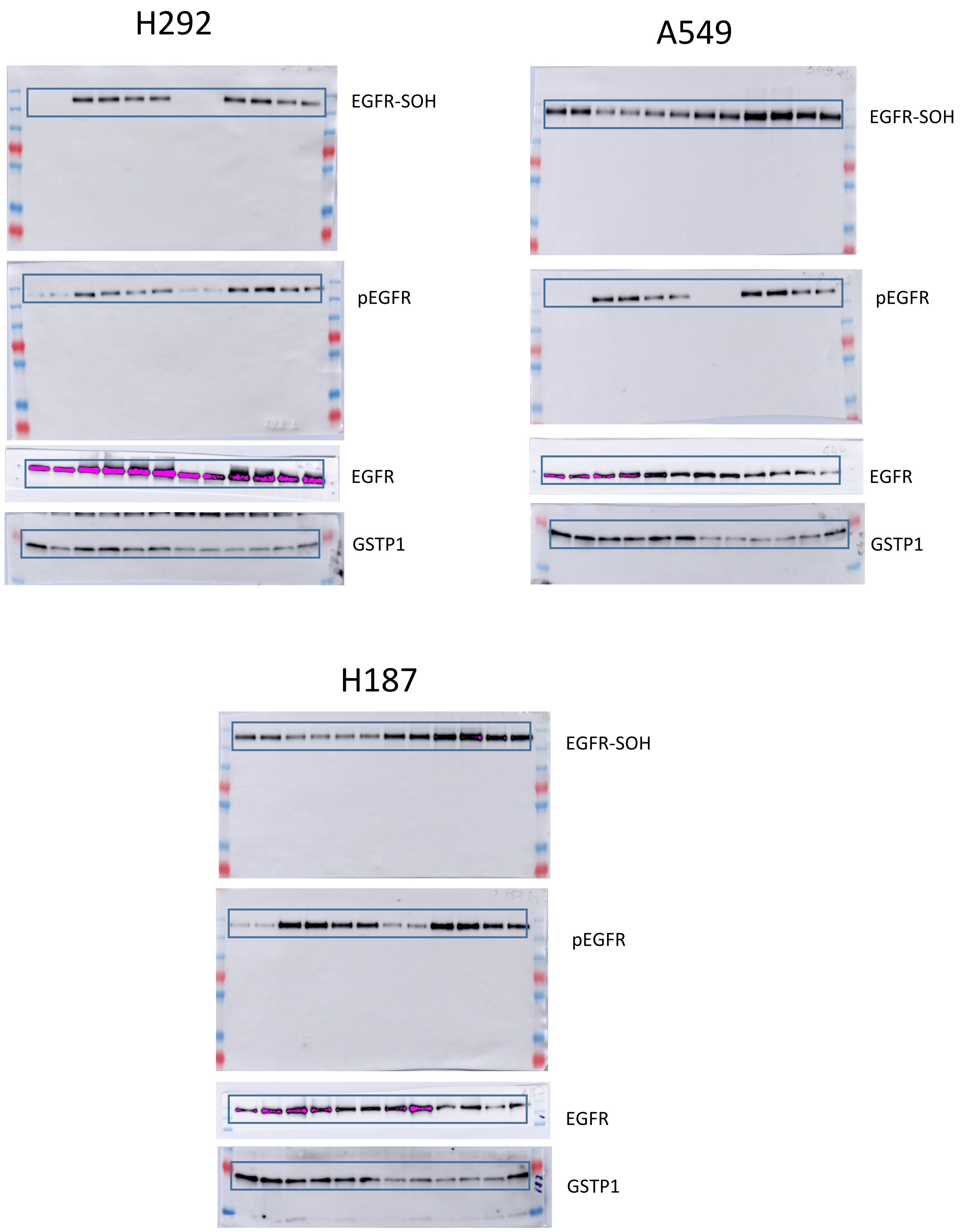

H292
EGFR-SOH
pEGFR
EGFR
GSTP1
A549
EGFR-SOH
pEGFR
EGFR
GSTP1
H187
EGFR-SOH
pEGFR
EGFR
GSTP1

Fig 4b

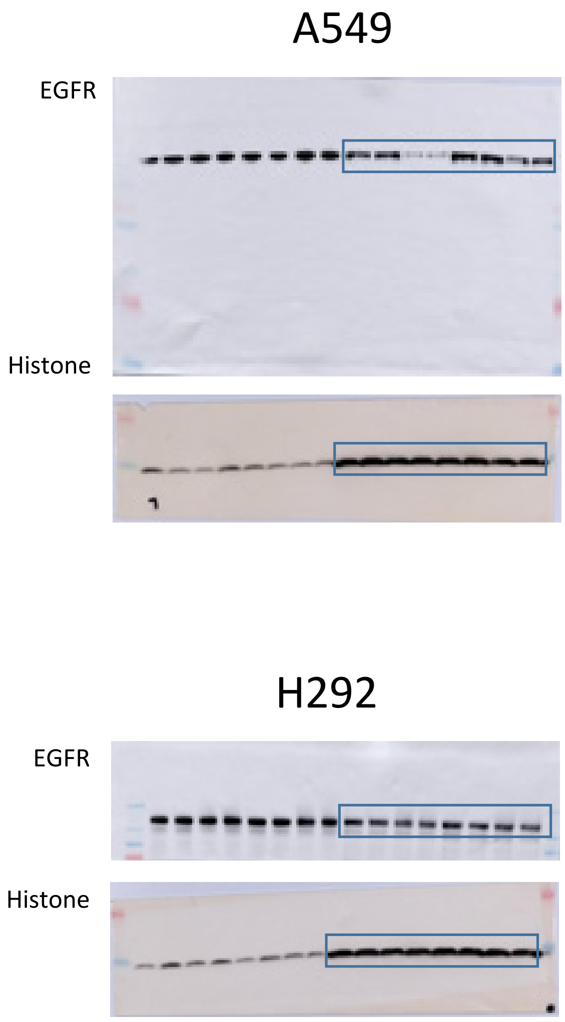

Uncropped western blots for Supplementary Figures

Fig S2

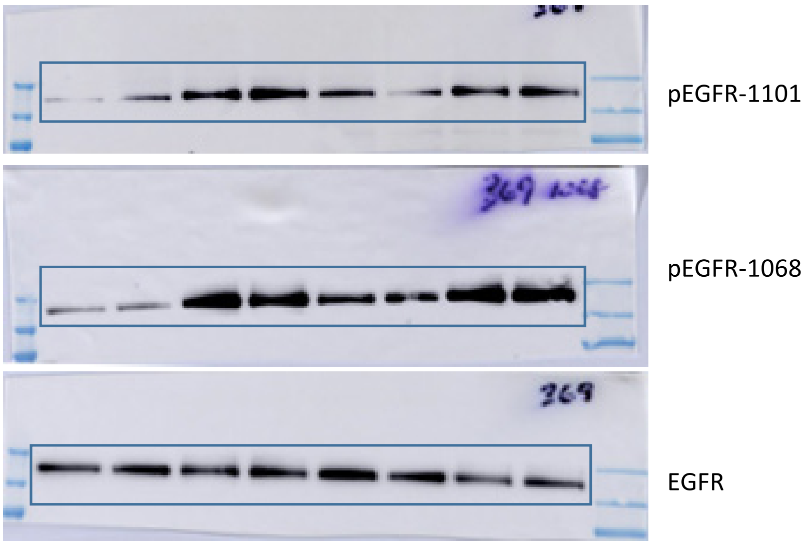

Fig S3

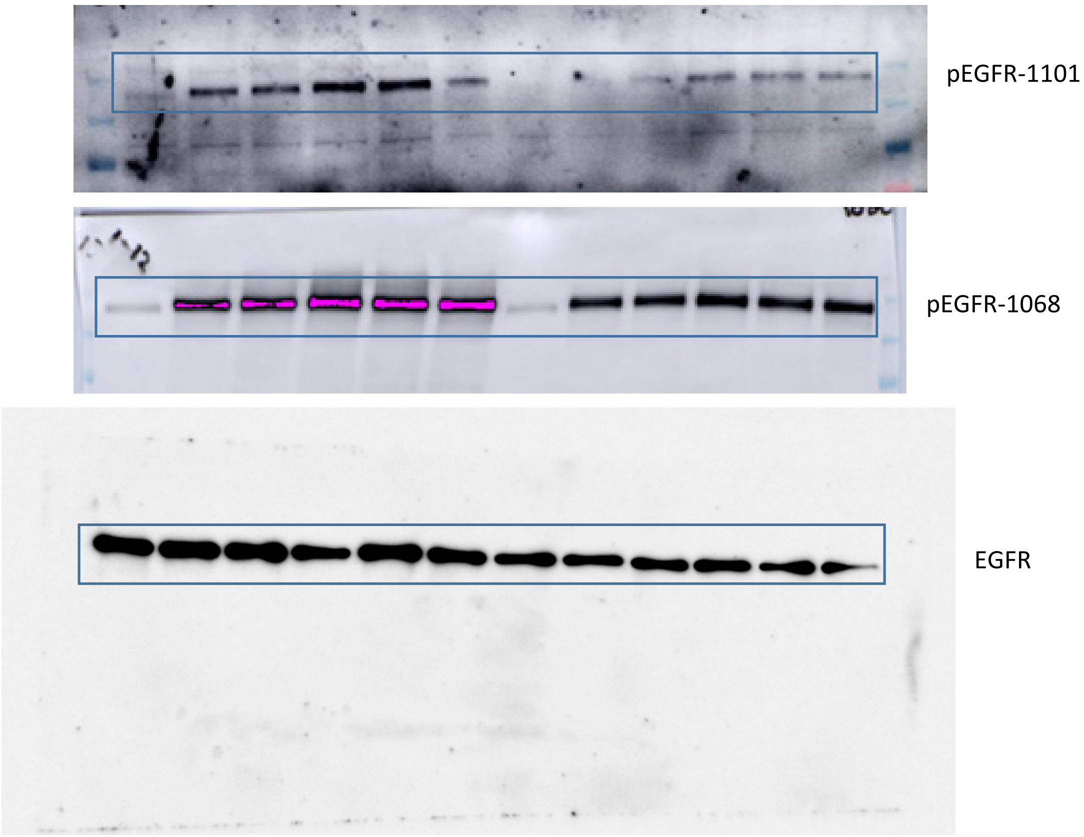

Fig S7A

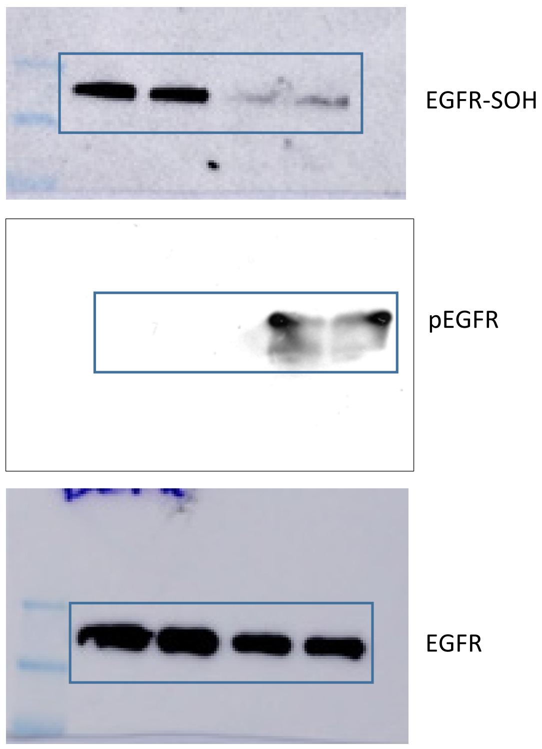

Fig S7B

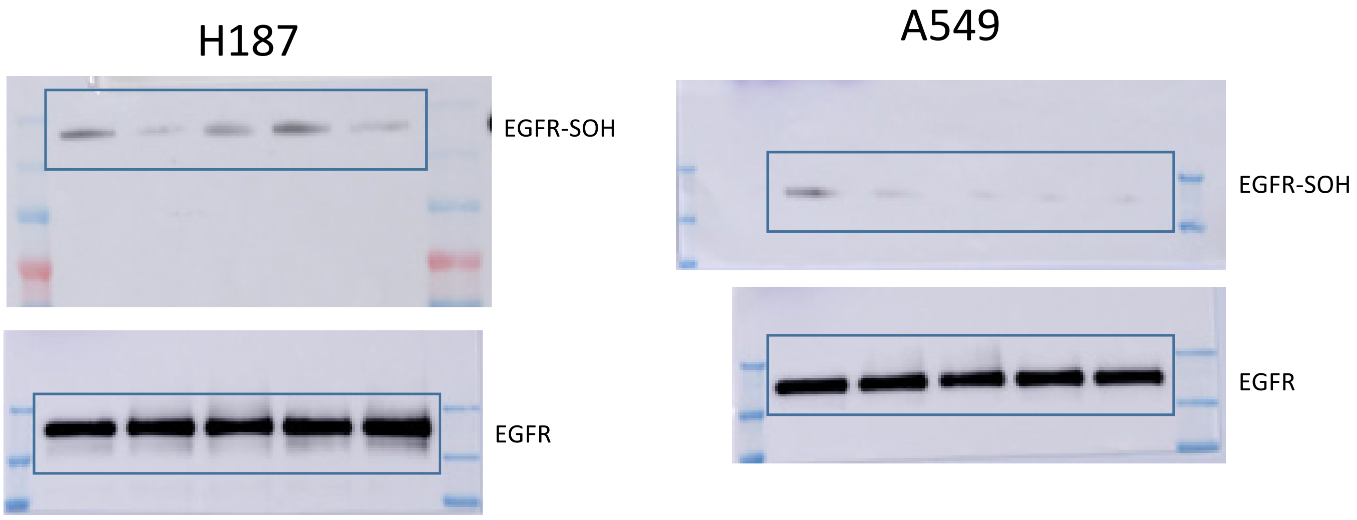

Fig S9

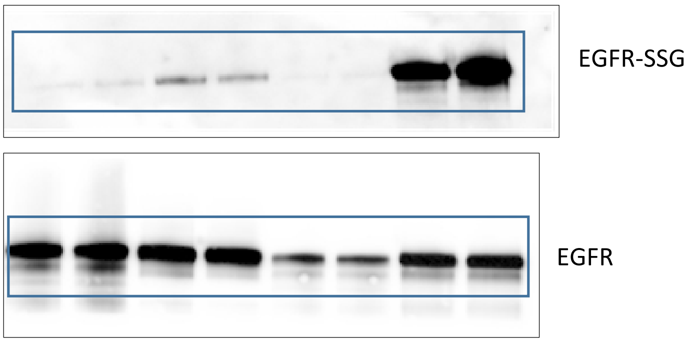

Fig S10

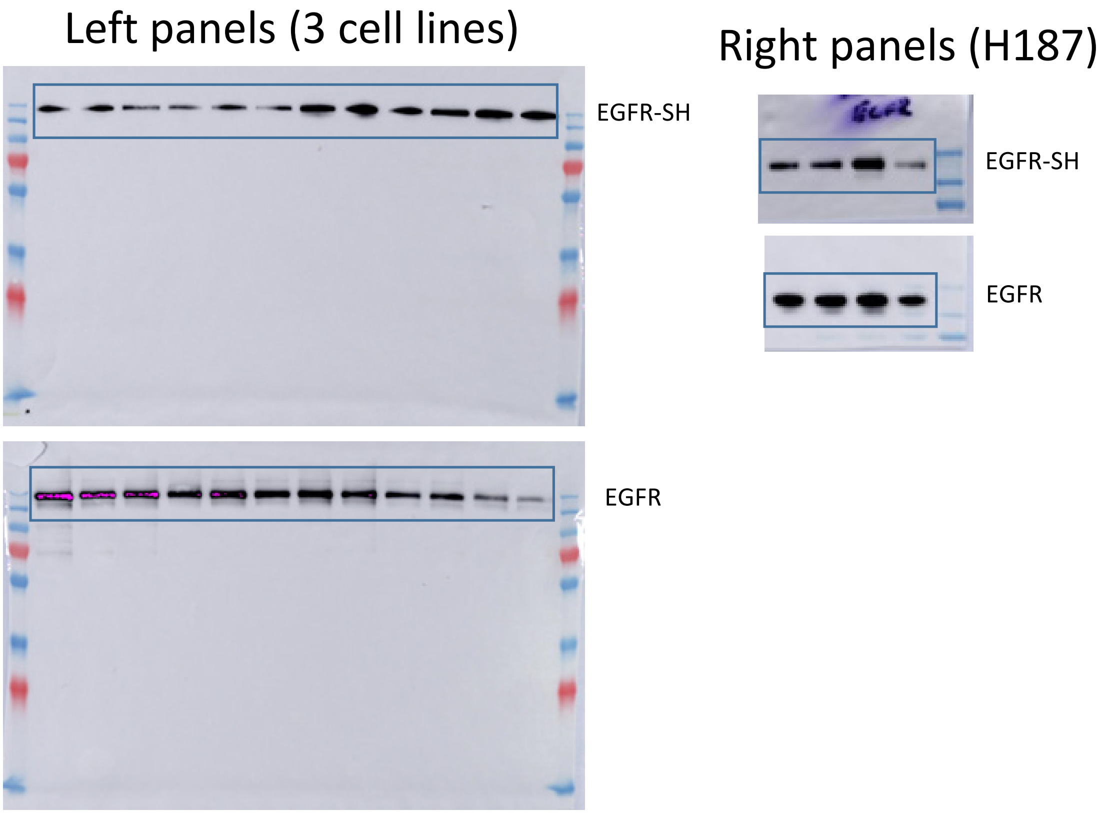

Fig S11

A549

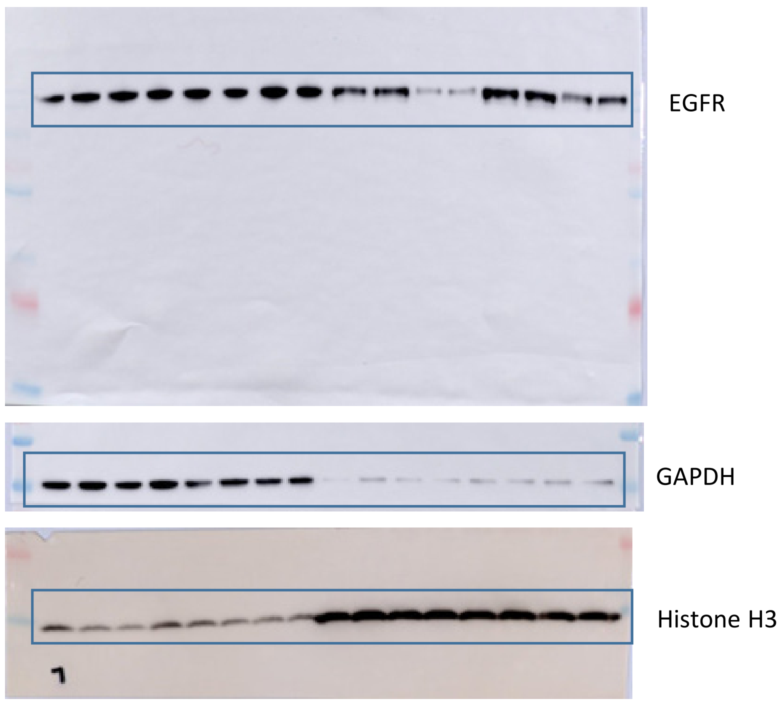

H292

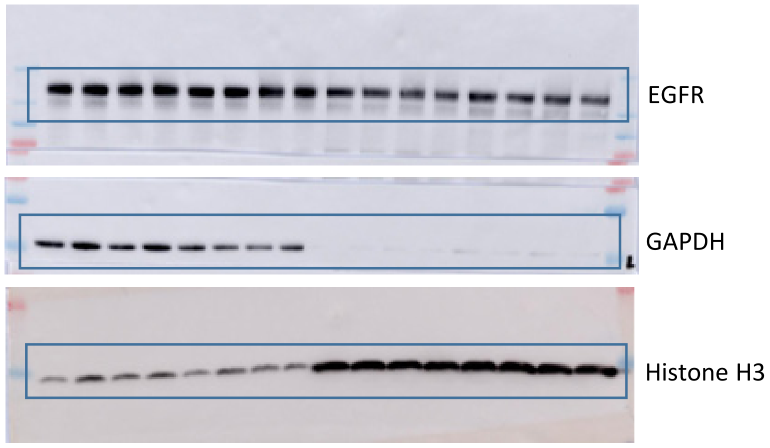

Supplement: Supplementary file 1 — Supplementary Information [file 41598_2019_41395_MOESM1_ESM.pdf]
